# Liquid-like cationic sub-lattice in copper selenide clusters

**DOI:** 10.1038/ncomms14514

**Published:** 2017-02-20

**Authors:** Sarah L. White, Progna Banerjee, Prashant K. Jain

**Affiliations:** 1 Department of Chemistry, University of Illinois at Urbana-Champaign, CLSL A, 601 South Goodwin Avenue, Urbana, Illinois 61801, USA; 2 Department of Physics, University of Illinois at Urbana-Champaign, Loomis Laboratory, 1110 West Green Street, Urbana, Illinois 61801, USA

## Abstract

Super-ionic solids, which exhibit ion mobilities as high as those in liquids or molten salts, have been employed as solid-state electrolytes in batteries, improved thermoelectrics and fast-ion conductors in super-capacitors and fuel cells. Fast-ion transport in many of these solids is supported by a disordered, ‘liquid-like' sub-lattice of cations mobile within a rigid anionic sub-lattice, often achieved at high temperatures or pressures via a phase transition. Here we show that ultrasmall clusters of copper selenide exhibit a disordered cationic sub-lattice under ambient conditions unlike larger nanocrystals, where Cu^+^ ions and vacancies form an ordered super-structure similar to the bulk solid. The clusters exhibit an unusual cationic sub-lattice arrangement wherein octahedral sites, which serve as bridges for cation migration, are stabilized by compressive strain. The room-temperature liquid-like nature of the Cu^+^ sub-lattice combined with the actively tunable plasmonic properties of the Cu_2_Se clusters make them suitable as fast electro-optic switches.

## Abstract (web summary)

Copper selenide is super-ionic only at high temperatures. Here, the authors discover room temperature super-ionic behaviour in ultrasmall clusters of Cu_2_Se—but not in its larger or bulk forms—owing to an unusual liquid-like cationic sub-lattice, in which octahedral sites are stabilized by size-dependent compressive strain.

Nanoscience is rife with examples of nanosized crystals displaying unique optical, electronic, chemical or structural properties not found in their bulk counterparts. For instance, in semiconductor nanocrystals (NCs) smaller in size than the Bohr excitonic radius, quantum confinement of carriers leads to discretization of energy levels and size-dependent excitonic transition energies and band-gaps^1^,^2^. Depression of solid-to-liquid melting points^3^,^4^ as well as solid-to-solid phase transition temperatures^5^,^6^,^7^,^8^ is also observed in small semiconductor and metal crystallites due to the increased contribution of surface energy to the total internal energy. An increase in interface-to-volume ratio in ionic materials, through reduction in size or nanostructuring of interfaces, has been found to reduce the formation energies of defects and thereby increase ionic transport^9^,^10^. Other nanoscale size effects in the thermodynamics and kinetics of pressure-induced structural phase transitions^11^ and chemical transformations^12^,^13^ are also known.

We investigate the unique structural and physical behaviour at an ultrasmall size of the semiconductor Cu_2_Se. Cu_2_Se is a solid with a peculiar ionic structure: the smaller Cu^+^ ions (eight or fewer per unit cell) have access to a much greater number of crystallographic sites within a rigid cage formed by the significantly larger Se^2−^ anions^14^. The large number of vacant sites available for Cu^+^ hopping is a primary factor in the manifestation of super-ionic transport in this solid. However, in its low temperature (LT) β phase, the vacancies are ordered and Cu^+^ ions are localized at the lowest-energy interstitial sites within a lower symmetry pseudo-cubic Se^2−^ sub-lattice^14^. Ionic transport in this form is rather limited. Above ca. 400 K, there exists a high temperature (HT) α phase of Cu_2_Se, in which the Cu^+^ ions form a disordered, liquid-like sub-lattice. The Cu^+^ ions are freely mobile between vacant and occupied sites within the immobile, face-centered cubic (fcc) Se^2−^ sub-lattice. This mobile Cu^+^ network supports Cu^+^ diffusivities (10^−5^–10^−4^ cm^2^ s^−1^) as high as those of liquids or molten salts and resulting ionic conductivities of 1–2 Ω^−1^ cm^−1^ (at 670 K), three orders of magnitude larger than the room temperature value^14^. This super-ionic behaviour is promising for replacing liquid electrolytes of batteries with solid-state ion conductors, developing fast-ion conductors for fuel cells and enhancing *zT* values for thermoelectric transport. However, the need for high temperatures can be limiting in these applications. In other closely linked systems, high pressures can alternatively be employed to achieve a super-ionic phase^15^.

Here we show that in the form of ultrasmall clusters, Cu_2_Se exhibits a disordered, liquid-like Cu^+^ sub-lattice under ambient conditions of temperature and pressure. We find that the mobile Cu^+^ network is linked to a unique cationic sub-lattice structure in the clusters that is remarkably different not just from the bulk form of the solid but also from larger NCs due to the effect of high compressive strain in the clusters. The preparation and subsequent study of this cluster form of Cu_2_Se is facilitated by cation exchange transformation of CdSe, which is available in the form of monodisperse, zinc blende-like clusters of ca. 2 nm size. For studying the comparative effect of crystallite size on room-temperature ionic structure, larger NCs are also prepared from size-controlled CdSe NCs using the same cation exchange method. The room-temperature super-ionic nature of Cu_2_Se clusters, combined with their actively tunable plasmonic properties, also demonstrated here, make them candidate materials for ultrafast electro-optic switching^16^.

## Results

### Synthesis and characterization of Cu_2_Se clusters

**Figure 1:**
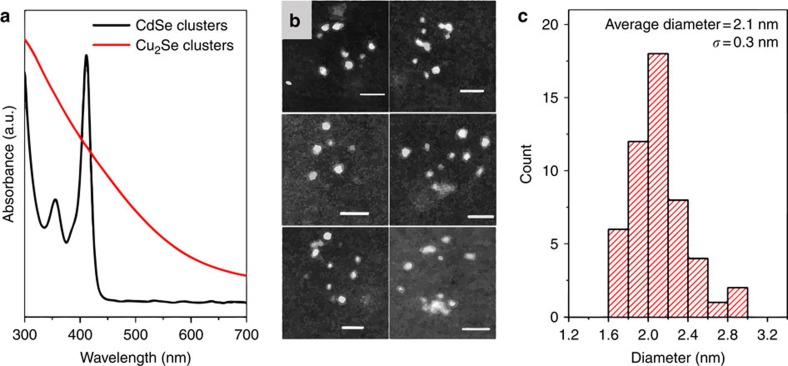
Synthesis and characterization of ultrasmall Cu_2_Se clusters. (**a**) The excitonic absorption spectrum of CdSe clusters (black curve) shows a narrow excitonic band at 406 nm, which on cation exchange with Cu^+^ ions is lost and a featureless band-to-band absorption of the indirect bandgap semiconductor Cu_2_Se appears (red spectrum). (**b**) HAADF-STEM images of Cu_2_Se clusters after exchange show that cation exchange of CdSe clusters results in discrete Cu_2_Se clusters with an average diameter of 2.1 nm±0.3 nm (s.d.) as shown by the (**c**) size histogram. Scale bars correspond to 10 nm.

Ultrasmall Cu_2_Se clusters were prepared by solution-phase cation exchange of magic-sized CdSe clusters with Cu^+^ (see ‘Methods' section). Thermodynamically stable, single-sized (ca. 2 nm) clusters of CdSe, popularized by the work of Kasuya and coworkers^17^, have been known to be formed in colloidal syntheses^18^,^19^. Their single-sized nature is manifested in their narrow 1S_h_–1S_e_ excitonic absorption peak at 406 nm (Fig. 1a), which shows lack of inhomogeneous broadening. Due to the topotactic nature of cation exchange, the anionic framework is preserved in the cation exchange process^20^,^21^, yielding ultrasmall Cu_2_Se clusters of similar ca. 2 nm size and fcc Se sub-lattice arrangement as the initial zinc blende CdSe template. The exchange process can be monitored by absorption spectroscopy (Fig. 1a). On addition of an excess of Cu^+^ to a solution of CdSe clusters, the narrow 1S_h_−1S_e_ excitonic absorption peak of CdSe is replaced by a featureless, band-edge absorption of the near-infrared (NIR) band-gap Cu_2_Se. Completion of exchange to yield the Cu_2_Se phase was verified by measurement of the elemental composition (Supplementary Fig. 1) by scanning transmission electron microscopy/energy dispersive spectroscopy (STEM/EDS). The Cu:Se ratio was found to be ca. 2:1, whereas the Cd signal was at the noise level of the EDS measurement, indicating little to no remnant Cd. High-angle annular dark field scanning transmission electron microscopy (HAADF-STEM) of the cation exchange product showed presence of discrete Cu_2_Se domains 2 nm in size (Fig. 1b,c and Supplementary Fig. 2).

### Cu_2_Se clusters sustain Cu vacancies and LSPRs

**Figure 2:**
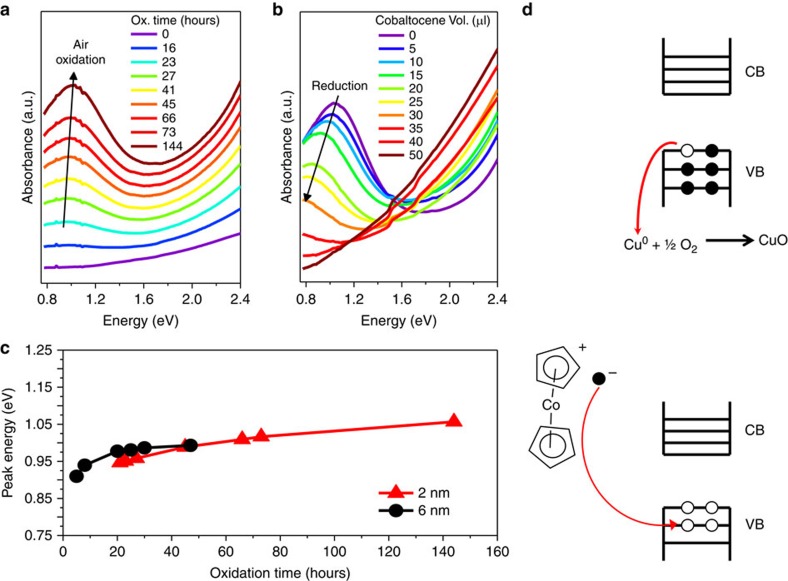
Cu_2−*x*_Se clusters display tunable LSPRs. (**a**) As Cu_2_Se clusters are oxidized by O_2_ in air, an LSPR absorption band appears in the NIR region, resulting from the creation of holes in the valence band of Cu_2_Se. The hole concentration in the clusters increases with increasing degree of oxidation, resulting in a blue-shift of the LSPR band. (**b**) Treatment of the oxidized clusters with the reducing agent cobaltocene results in the annihilation of the holes, leading to a gradual red-shift and suppression of the LSPR band. (**c**) In the process of oxidation, the LSPR peak energy of the Cu_2_Se clusters saturates to a value close to that for larger 6 nm Cu_2_Se NCs. (**d**). Schematic of the chemical oxidation and reduction of Cu_2_Se clusters. CB and VB indicate the conduction band and valence band of a cluster respectively. (top) An electron is extracted from the cluster by the process of copper oxidation, resulting in the formation of a hole in the VB. (bottom) On treatment by the reducing agent, cobaltocene, an electron from cobaltocene is injected into the cluster, annihilating a VB hole.

The clusters, synthesized at this size were found to exhibit a hallmark property of Cu_2_Se. It is known that copper chalcogenides, including Cu_2_Se, are stable in considerably Cu-deficient stoichiometries, Cu_2-*x*_Se (0≤*x*≤0.25), and therefore can be heavily p-type doped^22^,^23^,^24^,^25^,^26^. The stoichiometry and associated doping level can be tuned by oxidation/reduction. Similar to larger copper chalcogenide nanostructures^22^,^23^,^24^,^25^,^26^, as shown here for 6 nm Cu_2_Se NCs in Supplementary Fig. 3, upon exposure to O_2_, Cu^0^ is removed from the clusters in the form of copper oxide, creating Cu deficiency in the lattice (Fig. 2d). The sub-stoichiometry contributes hole carriers to the valence band, leading to the emergence of a localized surface plasmon resonance band (LSPR) in the NIR region of the absorption spectrum (Fig. 2a)^22^. With increasing oxidation levels, there is an increase in the peak energy *ω*_sp_ of the LSPR band (Fig. 2c), which is reflective of the increase in the free hole carrier concentration *N* as *ω*_sp_*α* 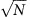. In this regard the clusters show behaviour similar to that of the larger 6 nm NCs, even though the oxidation kinetics appear to be slower for the 2 nm clusters (Fig. 2c). The LSPR peak energy of the clusters saturates to a value of 1.06 eV on prolonged oxidation, only marginally higher than the saturation peak energy of 0.99 eV for the 6 nm Cu_2_Se NCs and the value of 1 eV from Manna and coworkers^23^. The difference is only 7% and could be due to increased carrier confinement in the clusters, known to cause a blue-shift^1^,^27^ or due to differences in the local medium refractive index presented by the ligand shell. The similarity of LSPR peak energies for the two sizes suggests that the saturation hole concentration in the clusters is of the same magnitude (within 14%) as that in the larger NCs and is ca. 4 × 10^21^ cm^−3^ as estimated before^22^ for a 1 eV LSPR peak energy. The latter doping level corresponds to a stoichiometry in the range of Cu_1.8_Se commonly found for this semiconductor. The Cu_2_Se clusters synthesized here also show reversible tunability of the doping level and the LSPR^23^,^28^. On exposure of the oxidized clusters to a reducing agent such as cobaltocene, electrons are injected back into the Cu_2−*x*_Se clusters, the hole carriers are progressively annihilated, and the NIR LSPR band red-shifts while decreasing in absorbance (Fig. 2b). The LSPR band can be fully suppressed, indicating a return to the initial stoichiometric or nearly-stoichiometric form with low doping levels. The ability to be self-doped, the NIR plasmonic nature, and active plasmonic tunability of the Cu_2_Se clusters can be particularly attractive for electro-optic switching. Such switching can be rapid given the ultrasmall size, which favors short nanometer-scale diffusion lengths for Cu^+^.

### Lack of vacancy ordering in Cu_2_Se clusters

**Figure 3:**
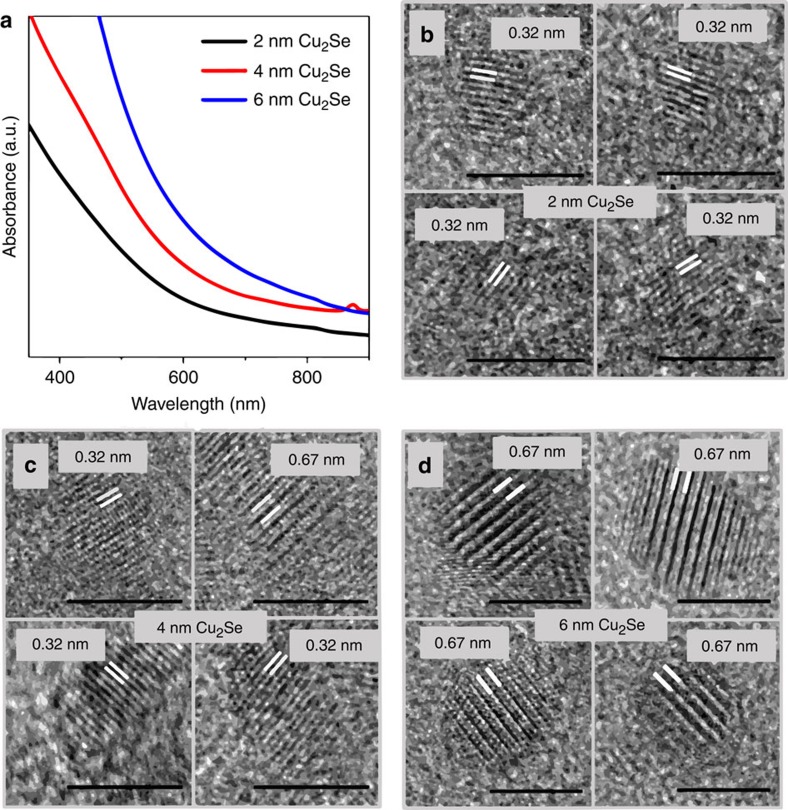
Superstructural vacancy ordering in Cu_2_Se NCs. HRTEM images of four representative Cu_2_Se NCs at each size: 2, 4, and 6 nm regime are shown in **b**, **c**, and **d**, respectively. Scale bars indicated correspond to 5 nm. Absorption spectra of the corresponding samples are shown in **a**. Vacancy ordering, characterized by a double lattice spacing of 0.67 nm along <111>, is evident in most 6 nm NCs and a fraction of 4 nm NCs; whereas such ordering is absent in 2 nm NCs, where a regular lattice spacing of 0.32 nm along <111> is seen. Image analysis was performed on ca. 30 NCs at each size and the percentage of NCs displaying superstructures is listed in Table 1. All images used for analysis are shown in Supplementary Figs 4–6.

Vacancy ordering below the order–disorder transition (ca. 400 K) temperature is another well-established hallmark of bulk Cu_2_Se (refs 29, 30, 31), which we studied in the clusters and the larger NCs (Fig. 3) using high-resolution transmission electron microscopy (HRTEM). The unit cell structures and the Cu^+^ sub-lattice arrangement in bulk Cu_2_Se have been extensively studied. It is known that even in the near-stoichiometric form of Cu_2_Se, vacant sites abound because the number of interstitial sites—8*c* tetrahedral, 32*f* trigonal and 4*b* octahedral locations per unit cell—available to the Cu^+^ ions is considerably larger than the number of Cu^+^ ions. In the ideal cubic anti-fluorite structure of the solid, the Se^2−^ anions occupy 4*a* sites forming an fcc sub-lattice with a lattice constant *a*_c_ of 5.85 Å, while the Cu^+^ cations fill all eight tetrahedral interstices within the fcc Se^2−^ cage. However, in practice, the LT β non-superionic phase has a defective anti-fluorite structure, wherein a fraction of the tetrahedral sites are vacant and the displaced Cu^+^ ions instead occupy trigonal sites^29^. Rather than arranging randomly, in the non-super-ionic phase these tetrahedral vacancies stack every four Cu^+^ layers along the <111> crystallographic axis creating a super-structure with a periodicity of 
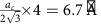
, which is twice the typical lattice spacing along the <111> direction^29^. As a result of vacancy ordering, the Se^2−^ sub-lattice is slightly distorted by few per cent with elongation along the stacking direction and contraction in the basal plane^29^.

Cu^+^ vacancy ordering is manifested in the form of a lattice fringe contrast pattern in HRTEM with an abnormally large 6.7 Å periodicity. In the HT super-ionic α-Cu_2_Se phase, the Cu^+^ ions are mobile between filled and vacant interstitial sites and the super-structural ordering is lost. The Se^2−^ sub-lattice is no longer distorted and restores its cubic arrangement. In this form, regular lattice fringes corresponding to an inter-Se planar spacing of 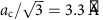 is seen along the <111> direction^29^,^30^. Thus, vacancy ordering is a signature of the LT β-Cu_2_Se non-super-ionic phase^29^,^30^,^31^. Most recently, Liu *et al*.^30^ used this super-structure contrast pattern in HRTEM to detect in bulk Cu_2_Se thermoelectric materials the transition from a non-super-ionic to a super-ionic, liquid-like Cu^+^ subsystem when the temperature was raised from ambient to 423 K, above the point of order–disorder transition.

In analogy to such observations on bulk Cu_2_Se, we employed HRTEM imaging to characterize at ambient temperature the presence of tetrahedral vacancy ordering and the resulting super-structure in the Cu_2_Se clusters and the NCs. Several NCs with high-resolution lattice patterns along <111> were analysed (Supplementary Figs 4–6), results of which are summarized in Table 1. Representative images of four NCs at each size are shown in Fig. 3. The 6 nm NCs (Fig. 3d) displayed a contrast pattern along <111> with a distance of 6.7 Å between bright fringes that represent Cu vacancy planes^29^. This contrast pattern arising from vacancy-ordered super-structure was observed in 80% of the 6 nm NCs, suggesting the existence of these NCs in the non-super-ionic phase. On the other hand, the 2 nm clusters (Fig. 3b) displayed a complete lack of super-structure, quite unlike 6 nm NCs or bulk Cu_2_Se at ambient temperature. Lattice fringes along <111> with a 3.2 Å periodicity of adjacent Se planes were seen, characteristic of the super-ionic phase with a mobile, disordered Cu^+^ sub-lattice. Interestingly, at the intermediate size of 4 nm, super-structural ordering was observed in only 10% of the NCs. Thus, as the crystallite size is reduced from 6 to 2 nm, HRTEM shows an increase in the statistical prevalence of a disordered Cu^+^ sub-lattice with no super-structural ordering. The results may be explained by a simple size-dependent reduction in the order–disorder transition temperature *T*_c_ such that the transition temperature is below ambient for the 2 nm size and above ambient for the 6 nm case. The depression of the phase transition temperature in nanosized domains of super-ionic solids like Cu_2_S, AgI and Ag_2_Se has been reported previously^5^,^7^,^32^. Invoking such an effect would imply that the 2 nm clusters are effectively in the HT α-Cu_2_Se phase at ambient temperature. From differential scanning calorimetry (DSC) measurements (Supplementary Fig. 7), indeed, the (ensemble-averaged) order/disorder transition temperature *T*_c_ for the clusters was found to be below room temperature (10 °C/−2 °C heating/cooling), significantly depressed compared to the temperature of 413 K or 140 °C reported for bulk Cu_2_Se (ref. 31).

### Unusual cationic sub-lattice structure of Cu_2_Se clusters

**Figure 4:**
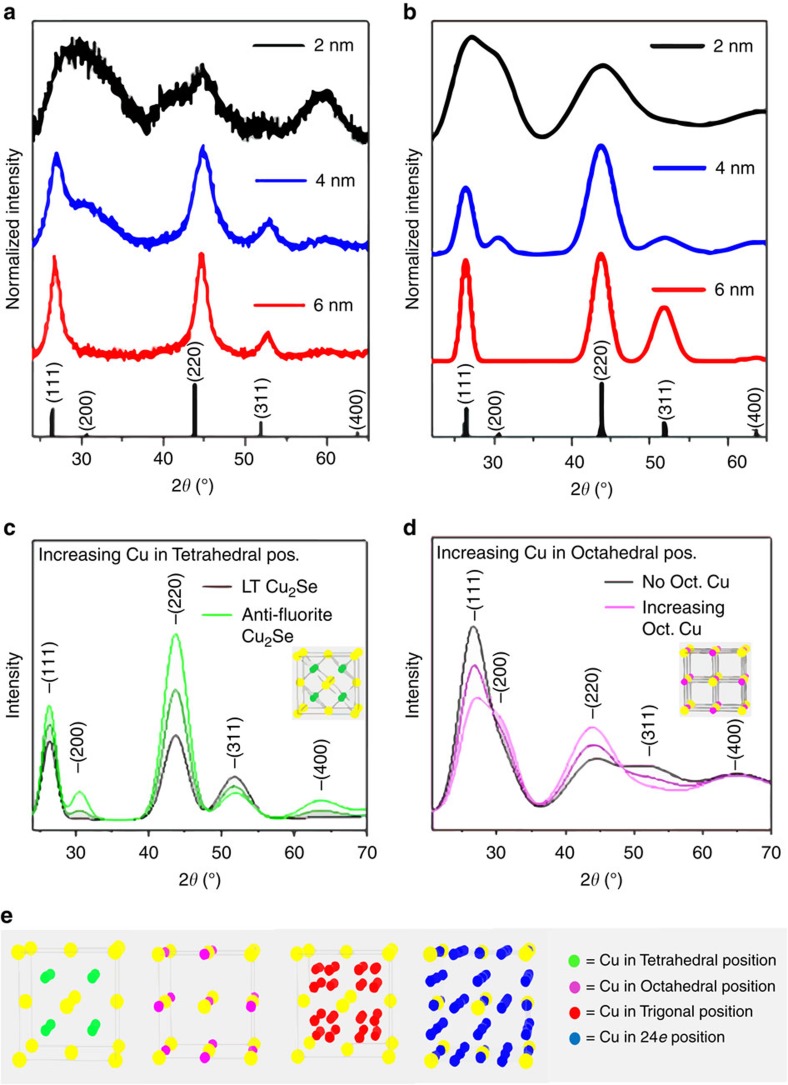
PXRD and cationic sub-structure. (**a**) Experimental and (**b**) simulated PXRD patterns of 2 nm clusters, 4 nm NCs and 6 nm NCs. The experimental pattern for the 6 nm NCs matches that of the LT phase of Cu_2_Se simulated on the basis of parameters from an established crystal structure. The {200} reflection missing in the pattern of 6 nm NCs is present in the pattern of 4 nm NCs. For 2 nm clusters, the {311} reflection is suppressed and the {200} reflection is strong. (**c**) Starting from the LT Cu_2_Se phase (black curve), the series of simulated PXRD patterns show that the progressive removal of Cu^+^ occupancy from trigonal positions and their placement in tetrahedral positions results is an increase in the intensity of the {200} reflection. (**d**) Starting from a structure in which all Cu^+^ occupancy is in tetrahedral and 24*e* positions (black curve), the series of simulated PXRD patterns shows that the progressive removal of Cu^+^ occupancy from 24*e* positions and their placement in octahedral positions results in a suppression of the {311} reflection and an increase in the {200} reflection intensity. Note, the peak at 2*θ*=59° is a background reflection from the sample holder (see Supplementary Fig. 8). (**e**) Schematic of different types of cationic sites within the Se^2−^ fcc cage, specifically tetrahedral, octahedral, trigonal and 24*e*.

However, further characterization of the Cu^+^ sub-lattice structure (Fig. 4 and Supplementary Figs 8 and 9) by powder X-ray diffraction (PXRD) suggests the presence of an unusual arrangement, which deviates considerably from the HT α phase of bulk Cu_2_Se. We compared the PXRD patterns of 2 nm Cu_2_Se clusters to those of 4 and 6 nm NCs (Fig. 4a). All three patterns closely matched that of cubic Cu_2_Se with a lattice parameter of *a*_c_=5.85 Å. However, there are notable differences as one goes from the 6 nm to the 2 nm Cu_2_Se: (i) the intensity of the {200} peak (2*θ*=30.5°) relative to that of the {111} peak (2*θ*=26.4°) increases, and (ii) the intensity of the {311} peak (2*θ*=51.8°) decreases. As we show below, these trends arise from differences between the cationic sub-lattice arrangements of the different size NCs, while the overall cubic structure of the unit cell, dictated by the rigid Se^2−^ arrangement, is mostly conserved.

We performed simulations using the programme Powder Cell which generates a PXRD pattern based on a structure factor calculation from unit cell parameters (Fig. 4b). Our simulation models employed an fcc Se sub-lattice. Known distortions from this close-packed arrangement of the anions, like those in the LT phase^29^, are minor enough to be undetectable in our PXRD data. In simulations, occupancies of the eight Cu^+^ ions in available interstitial sites: 4*b*, 8*c*, 24*e* and 32*f* (Fig. 4e) within the rigid fcc Se^2−^ cage were varied until the simulated PXRD pattern matched the experimental one for each size. In this manner, Cu^+^ sub-lattice structures were determined for 2, 4 and 6 nm Cu_2_Se. It must be noted that site occupancies determined are statistical: they do not imply a necessarily static distribution of Cu^+^, but reflect a dynamic average, which is why even a mobile arrangement with significant site-to-site hopping, can be characterized in such terms.

As per simulations, the experimental PXRD pattern of 6 nm Cu_2_Se agrees with an established structure for LT β Cu_2_Se, in which only a third of the Cu^+^ ions occupy 8*c* tetrahedral positions, whereas a majority occupy 32*f* trigonal positions^29^. The Cu_2_Se sub-lattice structure of 6 nm NCs is thus consistent with the presence of tetrahedral vacancies, which order in the LT phase. Comparison with occupancies of bulk Cu_1.8_Se (ref. 33) suggests that the filling of all eight tetrahedral sites in the stoichiometric solid (ideal anti-fluorite) would constitute a high-energy configuration due to Cu^+^–Cu^+^ repulsion. Occupation of other trigonal sites is therefore favoured at lower temperatures.

As the temperature is increased, the relative occupation of the tetrahedral sites increases^34^. In fact, in the HT phase, tetrahedral occupancy is significantly greater than trigonal site occupancy^35^, possibly due to the increased mobility of Cu^+^. Such an increase in tetrahedral occupancy (simulated in Fig. 4c) leads to the emergence of the {200} reflection and an increase in its intensity relative to the {111} intensity. In the anti-fluorite Cu_2_Se structure, the {200} reflections from the Se planes are cancelled out by {200} reflections from adjacent Cu planes, spaced from the Se planes by *a*_c_/4 layers. The cancelation is most effective when the tetrahedral sites are only half filled, that is, by four Cu^+^ ions to compensate for four Se^2−^ anions in the fcc anionic sub-lattice. When more of the tetrahedral sites are occupied by Cu^+^, a {200} reflection originates from the Cu–Cu inter-planar arrangement with a spacing of *a*_c_/2 units. When all eight tetrahedral sites are filled, the {200} reflection is the strongest. The change in the experimental PXRD in going from 6 nm to the 4 nm NCs is exactly in line with such a change in the Cu^+^ sub-lattice. In fact, the best match for the experimental PXRD pattern of the 4 nm NCs, which shows a distinct {200} peak, is the anti-fluorite structure, wherein all or most 8*c* tetrahedral sites are occupied and there is little to no occupancy in 32*f* trigonal sites. Thus, the increase in tetrahedral site occupation measured by PXRD goes hand-in-hand with the decrease in the propensity of tetrahedral vacancy ordering seen in HRTEM.

The experimental PXRD pattern for 2 nm clusters could not be reproduced using only *8c* tetrahedral and 32*f* trigonal site occupation. In this pattern, the {111} and {200} peaks are nearly equal in intensity, which could be simulated only by placement of Cu^+^ in 4*b* octahedral positions (Fig. 4d). The {311} peak is weak in intensity in the 2 nm clusters, which is also consistent with octahedral occupancy (Fig. 4d). Filling of octahedral sites with Cu^+^ leads to the emergence of {200} reflections associated with Cu–Cu and Se–Cu inter-planar arrangement with spacing of *a*_c_/2 units, leading to an overall {200} peak of strong intensity. On the other hand, the Cu planes arising from octahedral occupation, normal to the {311} direction, serve to cancel the {311} reflection from adjacent Se layers.

A fit to the experimental PXRD of 2 nm clusters was obtained by a structure with 28% tetrahedral occupancy, 18% octahedral occupancy and the remaining Cu^+^ in 24*e* interstitial sites (Supplementary Fig. 9). The significant occupancy in octahedral sites is quite striking, since these sites play an important role in the mechanism of fast-ion conduction in fcc solids, where a major mode of cation migration is along <111> through faces shared by tetrahedra and octahedra within the fcc unit cell^36^. Thus, octahedral sites (O) serve as bridging or intermediary sites for hopping between tetrahedral sites (T). But in α-Cu_2_Se, unlike in α-CuI (another common fcc super-ionic solid)^37^, the much greater number of Cu^+^ ions per unit cell and the consequently higher Cu^+^–Cu^+^ repulsion renders octahedral sites energetically unfavourable. While XRD and neutron diffraction studies suggest negligible occupation of 4*b* octahedral sites in LT β-Cu_2_Se phase, occupancy is only 5% even in the HT α-Cu_2_Se phase, whereas it is 15% in the clusters at ambient temperature.

If it were not for the infeasibility of octahedral occupation and the consequently high activation energy for -T-O-T- migration paths, α-Cu_2_Se would be much more ionically conductive than α-CuI due to twice the number of mobile cations in the former. The unusual sub-lattice structure of Cu_2_Se clusters combines the high cation density of Cu_2_Se and high octahedral occupation found in α-CuI (30% occupancy at 743 K). Moreover, this combination of attributes, desirable for fast-ion transport via -T-O-T- paths, is achieved in the clusters at room temperature. The statistical site occupancies determined from PXRD and the complete lack of superstructural ordering capture a dynamic cationic sub-lattice in the clusters where Cu^+^ ions are mobile (either via hopping or anharmonic thermal vibrations)^34^ over multiple tetrahedral and octahedral sites along <111> -T-O-T- conduction paths, at room temperature. Such a scenario is paraphrased by the liquid-like description of the Cu^+^ sub-lattice. Likewise, 24*e* site occupancy may suggest that their role as intermediary sites in tetrahedral-to-tetrahedral site hopping along <100>.

## Discussion

**Figure 5:**
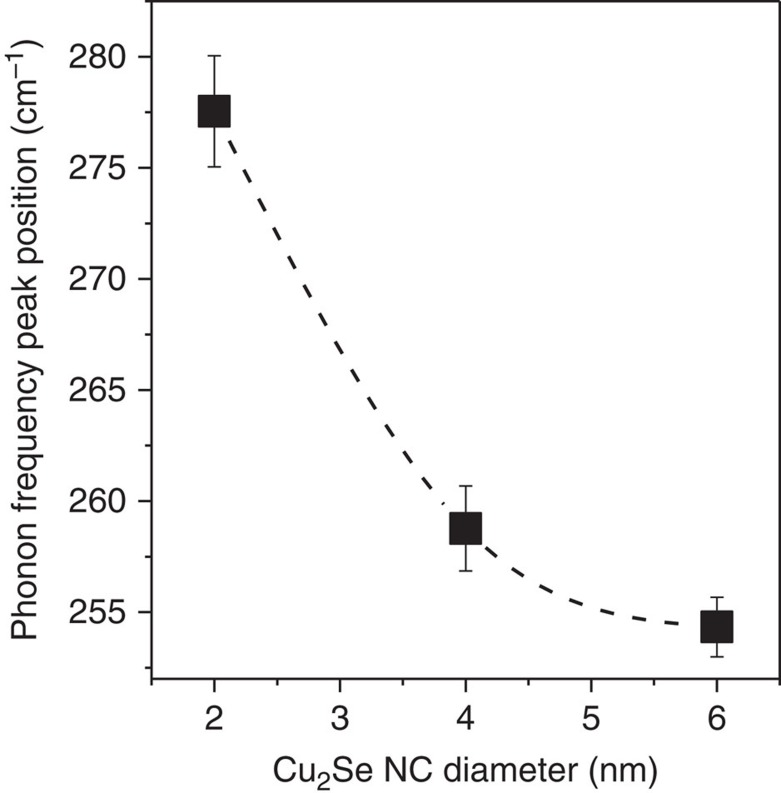
Compressive strain at ultrasmall size. The frequency of the LO phonon mode of Cu_2_Se increases from 6 nm to 4 nm to 2 nm Cu_2_Se NCs, indicating that the smallest NCs are considerably more compressively strained. Raman scattering spectra used for determination of the LO phonon mode frequency are shown in Supplementary Fig. 10. The LO phonon frequency plotted here is an average obtained from multiple measurements with the standard deviation (s.d.) represented by the error bar.

Whereas a combination of tetrahedral and octahedral site occupation is critical for barrier-free ion transport, these sites are weakly populated in bulk Cu_2_Se in the absence of thermal activation. As described before, it is energetically unfavourable to fill all 8*c* tetrahedral sites due to significant Cu^+^–Cu^+^ repulsion. Octahedral occupation is even less favoured due to heavy repulsion between an octahedral Cu^+^ and its four tetrahedral Cu^+^ neighbours^37^. How are octahedral sites then significantly populated in 2 nm clusters at room temperature? Taking a cue from the pressure-induced phase transition of binary cubic solids from tetrahedrally co-ordinated zinc blende structures to octahedrally co-ordinated rock-salt structures^38^, it may be hypothesized that the effect of cation repulsion may be countered by high pressure or compressive strain. Since our measurements are carried out at ambient pressure (in fact, the HRTEM is performed in vacuum), we explored the presence of compressive strain in the NCs. To determine the residual strain in the Cu_2_Se lattice, phonon scattering spectra were measured for NCs of all three sizes at room temperature (Supplementary Fig. 10). The A_1_ longitudinal optical (LO) phonon mode of Cu_2_Se (ref. 39) was detected in all three samples, but with a frequency ω that increased with decreasing NC size (Fig. 5). This mode hardening indicates the presence of a compressive strain in the NCs,^40^ which increases in magnitude with decreasing size. It must, however, be acknowledged that the LO phonon frequency can shift also due to the effect of phonon confinement in small crystallites, as shown by the classic work of Richter *et al*.^41^. The phonon confinement effect causes a red-shift of the phonon frequency, opposite in trend to the influence of compressive strain in NCs. These competing effects were discussed by Scamarcio *et al*. in CdS_1−x_Se_x_ NCs embedded in glass^42^, where it was shown that the effect of compressive strain on the phonon frequency more than overcomes the confinement effect. Thus, the compressive strain within a NC measured using the blue-shift of the phonon frequency serves as a lower limit; the actual compressive strain may be somewhat larger in magnitude. In fact, from the {111} peak in experimental PXRD patterns, the clusters were estimated to be compressively strained by 4.5% relative to the 6 nm NCs (Supplementary Fig. 11), although it must be acknowledged that the strain measured by PXRD has uncertainties due to the overlap and asymmetric broadening of peaks. Nevertheless, PXRD and phonon spectroscopy are qualitatively consistent in the finding of a compressive lattice strain in the clusters.

Such a size-dependent compressive strain in nanoparticles is a well-known effect of the high % of surface atoms of nanoparticles and their resulting propensity to undergo compression to reduce surface energy^40^, which is simply described by the Gibbs–Thomson relationship:


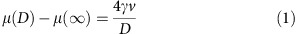


where *μ*(*D*) is the molar free energy of a NC of diameter, *D*, *μ*(∞) is the free energy in the bulk, *γ* is the surface tension and *v* is the molar volume. At smaller NC sizes, the surface energy term (r.h.s. in equation (1)) increases. To overcome the resulting increase in free energy, the NC undergoes a volumetric compression. For colloidal NCs, the degree of compressive strain is dictated not just by the size, but also by surface faceting and the nature of surface ligands, which influence *γ*: strongly passivating ligands can reduce the surface tension *γ*, thereby relieving some degree of strain caused by small size. The higher compressive strain measured in the 2 nm Cu_2_Se clusters relative to the larger NCs is a result of the smaller crystallite size of the former along with some contribution from differences in ligand passivation: the clusters are capped with trioctylphosphine (TOP), whereas the 4 and 6 nm NCs are capped with octylamine.

By using the relation^42^:


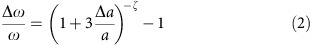


where *ζ* is the Grüneisen parameter for LO phonons assumed to have the common value of 1.1, we estimated that the compressive strain 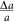 in the 2 nm clusters is 2.4% relative to the larger 6 nm NCs. The compressed lattice in the clusters can electronically stabilize Cu^+^ occupation in the six-coordinate octahedral sites. In the bulk Cu_2_Se lattice, a Cu^+^ occupying an octahedral site is at a distance of *a*_c_/2 from six Se^2−^ anions, but at a smaller distance of ✓3*a*_c_/4 from the other Cu^+^ ions occupying tetrahedral sites. Thus, the net dominant effect of Cu^+^–Cu^+^ repulsion destabilizes Cu^+^ occupation of an octahedral site in Cu_2_Se (ref. 37). In a compressed Cu_2_Se lattice, however, the bond distance between the octahedral Cu^+^ and the sixfold Se^2−^ neighbours is shorter, resulting in strengthened Cu–Se bonding that can potentially offset the Cu^+^–Cu^+^ repulsion. Expressed in terms of Pauling's rules of bonding in ionic crystals, tetrahedral co-ordination between Cu and Se is favoured in the typical Cu_2_Se lattice; but, octahedral co-ordination can become relatively favourable when the density is increased, as is the case for the compressed lattice (ca. 7% smaller unit cell volume) of the clusters. Using the bulk modulus of 85 GPa of the closely related Cu_1.5_Se (ref. 43) as an estimate for Cu_2_Se (for which a bulk modulus is not available), the compressive strain in the clusters, relative to 6 nm Cu_2_Se is equivalent to the application of a 2 GPa pressure, which is similar to the magnitude of pressure at which CdSe transforms from the four-co-ordinate wurtzite/zinc blende phase to the six-coordinate rock-salt phase with octahedral sites filled with cations^44^.

Since octahedral bridging sites play a critical role in Cu^+^ migration in Cu_2_Se, the energetic cost of octahedral occupation of Cu^+^ is likely to influence the activation energy, *E*_a_, of Cu^+^ sub-lattice disordering/melting. From phonon scattering measurements, we find that with decreasing NC size *D*, the lattice is under an increasing degree of compressive strain, which we postulate to result in an increasing stabilization of octahedral Cu^+^ occupation (as manifested by the crystallographic findings) and, consequently, a decreasing activation energy, *E*_a_, for cationic disorder. Such a size-dependent *E*_a_(*D*) can explain the results from HRTEM analysis (Table 1). Even at a specific NC size *D*, why is it that a sub-population of the NCs is in the ordered state, while the remaining is in the disordered, super-ionic state? This is because, in nanocrystalline samples, phase transition points are not necessarily sharp; rather they may be heterogeneously broadened. Thus, even at a temperature *T*>*T*_c_, the (ensemble-averaged) order/disorder temperature, the ordered phase may persist in a sub-population of NCs. The per cent sub-population of NCs in this frozen state, expected to vary as e^*Ea(D)/RT*^, is indeed found to decrease with decreasing NC size, *D* (Table 1). Specifically, for the 2 nm clusters, not only is the order/disorder transition temperature, *T*_c_, below room temperature, but a low activation energy for cationic disorder ensures the prevalence of a molten Cu^+^ sub-lattice across all NCs in the measured ensemble (Table 1).

In summary, we found that ultrasmall Cu_2_Se clusters exhibit a mobile, liquid-like Cu^+^ sub-lattice at room temperature, quite unlike larger NCs and bulk Cu_2_Se where such a super-ionic phase is seen at significantly higher temperatures. Possibly due to the effect of compressive strain, the clusters exhibit an unusual cationic sub-lattice structure, wherein energetically unfavourable sites in the conduction pathway are stabilized. NC size tuning of phase transition temperatures is well known, but the influence of NC size and associated strain on ionic structure and transport is an open area of investigation. The findings provide insight into the role of vacancies, cation-anion co-ordination, and nature of bonding in the achievement of fast-ion conduction, which may lead to broader design principles^36^ beyond the specific Cu_2_Se system studied here. For instance, high-pressure super-ionic phases have been predicted in other materials such as ice^45^. -T-O-T- pathways are important in solid-state lithium ion conduction^36^.

The properties of Cu_2_Se clusters make them promising for the fabrication of nanostructured conductors for solid-state electrolytes and ionic switches, which can be operated at room temperature. However, a near-term challenge will involve the achievement of fast-ion conduction through solids comprised of the room-temperature super-ionic phase of Cu_2_Se. The clusters will need to be assembled into mesoscopic or macroscopic solids, interfacial defects and ligands will need to be eliminated without modification of the nanocrystalline morphology or the crystallographic phase, and the ionic conduction will need to be characterized using alternating current (AC) impedance measurements, an effort currently underway in our laboratory.

The actively tunable plasmonic properties of the Cu_2_Se clusters can be exploited for electro-optic switching. Since, the switching ON/OFF of the optical resonance would rely on voltage-assisted in/out migration of a Cu^+^ ion from the cluster, the fast-ion transport characteristics of the system can be particularly advantageous. Lindenberg and coworkers^46^ have shown that in the super-ionic phase of Cu_2_S, Cu^+^ hopping between adjacent sites takes place on the 20 picosecond time-scale. Given that a small number of hops are sufficient for Cu^+^ to migrate into/out of a 2 nm cluster, ultrafast operation may be possible.

## Methods

### Synthesis of 2 nm CdSe clusters

The procedure for the synthesis of CdSe clusters was adapted from Yu *et al*.^19^. Cadmium acetate (0.20 mmol, 53.3 mg) and oleic acid (0.13 mmol, 41 μl) were added to a 25 ml three-neck flask. The flask was repeatedly purged with Ar to remove O_2_. Under Ar atmosphere, 5 g of TOP was added to the flask using a syringe and the reaction mixture was heated to 120 °C, and then subject to vacuum for 45 min. Then, the reaction mixture was brought to Ar atmosphere, the temperature was dropped to 100 °C, and a TOP-Se solution (0.05 mmol or 4 mg of selenium powder in 0.4 ml of TOP) was added to the flask. The temperature was raised to 120 °C and the reaction was allowed to proceed for 60 min. The solution was a light yellow colour. After synthesis, the clusters were washed repeatedly with toluene and methanol and dispersed in toluene.

### Synthesis of 4 nm CdSe NCs (zinc blende)

The procedure for synthesis of 4 nm CdSe NCs was adapted from Yang *et al*.^47^ Cadmium myristate (192 mg, 0.34 mmol) was added to a flask with 3.4 g, that is, 4.3 ml of octadecene (ODE). The reaction mixture was heated to 140 °C under Ar atmosphere. The solution was then cooled to 100 °C and subject to vacuum for 30 min. The solution was then put under Ar and cooled to room temperature. A solution of TOP-Se was prepared in a glove box by dissolving 13.4 mg of Se (0.17 mmol) in 126 mg of TOP (0.34 mmol). The TOP-Se solution was injected into the reaction flask. The temperature was raised to 210 °C, which occurred over ∼9 min. After reaching 210 °C, 48 mg of oleic acid in 0.5 ml ODE was injected into the flask to stabilize NC growth. Then the reaction proceeded at 210 °C for 50 min. After synthesis, the NCs were washed repeatedly with toluene and methanol and dispersed in toluene.

### Synthesis of 6 nm CdSe NCs (wurtzite)

The procedure for synthesis of 4 nm CdSe NCs was adapted from Carbone *et al*.^48^ Cadmium oxide (0.06 g), octadecylphosphonic acid (0.28 g) and trioctylphoosphine oxide or TOPO (3.0 g) were added to a 50 ml three-neck round-bottom flask. The flask was degassed under vacuum at 150 °C for 1.5 h. The flask was then put under Ar atmosphere and the temperature was gradually increased to 300 °C over the course of 2 h. While slowly ramping up the temperature up to 320 °C, the solution becomes optically clear. After reaching 320 °C, 1.8 ml of TOP was gradually injected into the mixture. A solution of TOP-Se was prepared in a glove-box by dissolving 0.6 g Se in 4.48 ml TOP and stirred at room temperature overnight. The temperature of the mixture in the flask was raised to 360 °C and then 0.45 ml of the TOP-Se solution was injected rapidly. After injection, the solution is heated at 360 °C for 70 s, or until the solution is a dark red colour, after which the heating mantle was removed. The NCs were washed repeatedly with toluene and methanol and dispersed in toluene.

### Synthesis of 6 nm CdSe NCs (zinc blende)

The procedure for synthesis of 6 nm CdSe NCs was adapted from Liu *et al*.^49^ 78.4 mg of Se and 15 ml of ODE were added to 50 ml three-necked round-bottom flask. The flask was heated to 100 °C and subject to vacuum for 30 min. At the same time, 266 mg of Cd(Ac)_2_ was added to a 25 ml round-bottom flask with 5 ml of oleic acid. The resulting solution was heated to 100 °C and subject to vacuum for 30 min. Both flasks were put under Ar again. The flask with the Cd salt was heated to 150 °C and the flask with Se was heated to 280 °C for 30 min. The Se solution turned yellow indicating the formation of a Se-ODE complex. Then the solution of Cd was quickly added to the other flask and the temperature was brought to 275 °C, after which the reaction was allowed to proceed for 40 min. After synthesis, the NCs were washed repeatedly with hexane and ethanol and redispersed in toluene.

### Ligand exchange with octylamine

For 4 and 6 nm CdSe NCs, it was found that an initial ligand exchange of the NCs with octylamine increased colloidal stability of the Cu_2_Se NCs produced from cation exchange. Before cation exchange, solutions of 4 and 6 nm CdSe NCs dispersed in toluene were mixed with ∼0.5 ml of octylamine, after which the NCs were washed with methanol and redispersed in toluene.

### Exchange with Cu^+^

All exchange reactions were carried out in an oxygen-free, moisture-free, Ar-filled glove box. CdSe NCs or clusters were dispersed in toluene. A solution of tetrakis(acetonitrile) copper(I) hexafluorophosphate ([(CH_3_CN)_4_Cu]PF_6_) in 10% v/v of methanol in acetonitrile was then added dropwise to the NC or cluster solution. The reaction mixture was stirred vigorously in the course of addition. Exchange was monitored by ultraviolet-visible absorption spectroscopy. In exchange reactions with 2 nm CdSe clusters, the Cu^+^ reagent was prepared with 1.5 equivalents of TOP per equivalent of [(CH_3_CN)_4_Cu]PF_6_ and was therefore added to the cluster solution in TOP-Cu^+^ form, a procedure that enhanced the colloidal stability of Cu_2_Se clusters formed from exchange.

### Oxidation of Cu_2_Se NCs and clusters

To the 2 and 6 nm Cu_2_Se NCs obtained from exchange, ∼50 μl of oleic acid was added for aiding colloidal stability. The samples were then washed with methanol and dispersed in toluene and sonicated for 20 min. Cu_2_Se NC or cluster colloids in vials were exposed to air with stirring to achieve oxidation. Small losses in solution volume resulting from evaporation over extended periods of time were counterbalanced by adding anhydrous toluene such that the solution height in the vial was maintained. Ultraviolet-visible-NIR (ultraviolet-vis-NIR) spectra in the 300–2,000 nm wavelength range were acquired at 1 h intervals at the onset of the oxidation. At later stages, spectra were acquired at longer time intervals. The colloids were sonicated before every spectral acquisition and a small aliquot of the colloid was extracted and diluted in an NIR transparent cuvette (Spectrocell) to prepare the sample for ultraviolet-vis-NIR spectroscopy. The spectral region with NIR absorption of toluene (ca. 1,640–1,740 nm) was removed manually from plotted spectra.

### Reduction of Cu_2_Se NCs and clusters

Oxidized Cu_2_Se NCs and clusters were reduced using a strong electron donor, cobaltocene. A cobaltocene solution containing ∼51 mg of cobaltocene in 4 ml toluene was prepared. Solution preparation and the reduction reaction were carried out inside the glove box because cobaltocene is a strong reducing agent that reacts quite rapidly with oxygen. The cobaltocene solution was added in increments of 5 μl–3.5 ml of the NC or cluster colloid in an NIR transparent cuvette. After each addition, the cuvette was tightly capped and the reaction mixture was allowed to stir for 40 min in the glove box, after which the cuvette was taken out of the glove box. An ultraviolet-vis-NIR spectrum was acquired in the wavelength range from 300–2,000 nm. The cuvette was brought back into the glovebox immediately after each spectral acquisition. Sealing the capped cuvette with black tape further helped minimize any air exposure. The spectral region with NIR absorption of toluene (ca. 1,640–1,740 nm) was removed manually from plotted spectra.

### STEM/EDS measurements

Elemental analysis was carried out on each of the three sizes of Cu_2_Se NCs by STEM/EDS. Cu_2_Se NCs for the measurements were prepared by cation exchange from zinc blende CdSe NCs. For the 4 and 6 nm CdSe NCs, before cation exchange, ligand exchange with octylamine was carried out to aid colloidal stability. Octylamine (200 μl) was added to a solution of the CdSe NCs dispersed in toluene, followed by washing of the NCs with methanol. The amine-passivated NCs were redispersed in toluene. The 2 nm CdSe clusters were cation exchanged without any prior ligand exchange step. For cation exchange, a solution of CH_3_CN)_4_Cu]PF_6_ in 10% v/v methanol in acetonitrile was added dropwise to the NC or cluster solution. The reaction mixture was stirred over the course of the addition. The exchanged NCs or clusters were cleaned by three successive methanol washing and centrifugation steps and finally redispersed in toluene. Ultraviolet-vis-NIR absorption spectra (350–1,600 nm) were recorded to ensure the completion of the exchange.

STEM/EDS was performed on a JEOL 2010F instrument operating at 200 kV. Samples were prepared by drop-casting NCs or clusters from solution onto ultrathin carbon 300-mesh Au grids from Ted Pella followed by repeated washing of the grid with methanol. A zero-background double-tilt holder was used for STEM/EDS measurements. EDS measurements were carried out over a wide-field (ca. 1 × 1 μm^2^) of NCs using an Oxford INCA 30 mm ATW detector. Data were collected for 500–800 s and the double-tilt holder was placed at a 10° elevation to maximize the signal. Elemental quantification was performed in the IXRF Iridium Ultra software, using the integrated intensities of the Cd L_α_ lines and the Cu and Se K_α_ lines in the EDS spectra. Atomic %s of Cu, Cd and Se, obtained from the IXRF Iridium Ultra software, were converted to atomic ratios by normalizing the Se content to 1. The Cu:Se:Cd atomic ratios are tabulated in Supplementary Fig. 1 along with EDS spectra for the three sizes.

### Phonon scattering measurements

Phonon scattering spectra (intensity versus Raman shift in the frequency range of 100–600 cm^−1^) of Cu_2_Se NC and cluster films on Si substrates were acquired at room temperature on a Horiba Raman confocal imaging microscope. The experiments were carried out using 532.07 nm laser excitation, without filters, and a high-resolution grating of 1,800 g mm^−1^ blazed at 500 nm. A 50 × long working distance objective and a spectrophotometer setting of 350 cm^−1^ were employed, resulting in a spectral resolution of ca. 2 cm^−1^.

Cu_2_Se samples for the measurements were prepared by cation exchange of zincblende CdSe NCs and clusters. For the 4 nm and the 6 nm zinc blende CdSe NCs, ligand exchange with octylamine was carried out to aid colloidal stability. 70 μl of octylamine was added to a solution of the NCs dispersed in toluene, followed by washing with methanol. The amine-passivated NCs were redispersed in toluene. The 2 nm CdSe clusters were cation exchanged without any prior ligand exchange step. For cation exchange, a solution of tetrakis(acetonitrile) copper(I) hexafluorophosphate ([(CH_3_CN)_4_Cu]PF_6_) in 10% v/v methanol in acetonitrile was then added dropwise to the NC or cluster solution. The reaction mixture was stirred over the course of the addition. The exchanged NCs or clusters were washed with methanol and redispersed in hexane. Ultraviolet-vis-NIR spectra were acquired in the wavelength range from 300–2,000 nm for all three sizes to determine that the exchange reaction was complete.

Before sample preparation, Si substrates were cleaned by sonication in methanol for 10 min, followed by drying in an oven at 120 °C for 15 min. About 40 μl of the Cu_2_Se NC or cluster solution was drop cast onto the Si substrate and dried to form a thin film. For each size: 2, 4 and 6 nm, four film samples were prepared and 1–3 spectra were acquired for each film. Only representative spectra are shown in Supplementary Fig. 10. The LO phonon peak position was determined from each one of the multiple spectra at each size. The averaged LO phonon peak frequency is plotted against NC size in Fig. 5. The standard deviation of the phonon peak position is shown as the error bar for each data point.

### Electron microscopy characterization

HRTEM and HAADF-STEM images were acquired on a JEOL 2010F operating at 200 kV. Samples were prepared by drop-casting NCs or clusters from solution onto an ultrathin carbon grid followed by repeated washing of the grid with methanol. Size analysis was performed by measuring particle size along the long axis on HAADF-STEM images using the software, ImageJ. Analysis of the lattice super-structure in each sample was performed using HRTEM images. HAADF-STEM of 4 and 6 nm Cu_2_Se NCs are shown in Supplementary Fig. 2.

### PXRD

PXRD patterns were collected on a Rigaku Miniflex 600 powder X-ray diffractometer operated at full power (40 kV–15 mA) with Cu K_α_ radiation wavelength (1.54 Å). Data were collected in reflection mode in the 2*θ* range of 15°–65° using a step size of 0.04° with scans running for 2–3 h. Samples were prepared by drop-casting NCs or clusters from solution into a thick film on a zero-background quartz substrate.

### PXRD simulations

Simulated PXRD patterns were generated using the programme PowderCell, which performs a structure factor calculation using lattice parameters and atomic positions as input parameters. Input parameters for each simulated pattern are tabulated (Tables 2, 3, 4). All simulated patterns include Debye-Scherer broadening corresponding to the finite crystallite size of 2, 4 and 6 nm, respectively.

### DSC measurements

DSC measurements were carried out on a DSC Q20 V24.10 Build 122 instrument. Before collection of a thermogram, each sample was subject to heating run from −30 to 200 °C. We have found that this procedure removes ligands from the NC surface (oleic acid, remnant phosphines and so on), thereby eliminating peaks related to ligand desorption that otherwise show up in the thermogram and complicate the analysis. For ensuring reproducibility, at each size, thermograms (cooling followed by heating) were measured for two separate samples. For atleast one sample at each size, the DSC scan was run twice and confirmed to be repeatable.

Cu_2_Se clusters for the measurement were prepared by cation exchange of CdSe clusters. The 2 nm CdSe clusters were cation exchanged by adding a solution of (CH_3_CN)_4_Cu]PF_6_ in 10% v/v methanol in acetonitrile dropwise to the cluster solution. The reaction mixture was stirred over the course of the addition. The exchanged clusters were washed with methanol multiple times and redispersed in toluene followed by drying under Ar in a glovebox. The dried powder, weighing a few mg, was pressed into a T-Zero aluminium pan for the DSC measurements.

The larger size Cu_2_Se NCs were prepared following the procedure of Deka *et al*.^50^ After preparation, the colloidal solution was dispersed in toluene and subjected to selective centrifugation to extract NCs in the desired size range. The NCs were washed twice with ethanol and then dispersed in toluene. An absorption spectrum was acquired in the vis-NIR range (400–1,600 nm) to confirm the presence of an LSPR typical of Cu_2_Se NCs. HRTEM imaging was also performed on a JEOL 2010F instrument operated at 200 kV with a 0.5-nm size beam to ensure a nanocrystalline morphology. For DSC measurements, the NC solution was centrifuged to obtain a pellet, which was then dried to obtain a few mg of solid. The solid was pressed into a T-Zero aluminium pan for DSC measurements.

### Data availability

The data that support the findings of this study are available from the authors on reasonable request.

## Additional information

**How to cite this article:** White, S. L. *et al*. Liquid-like cationic sub-lattice in copper selenide clusters. *Nat. Commun.* **8**, 14514 doi: 10.1038/ncomms14514 (2017).

**Publisher's note:** Springer Nature remains neutral with regard to jurisdictional claims in published maps and institutional affiliations.

## Supplementary Material

Supplementary Information Supplementary Figures and Supplementary References.

## Figures and Tables

**Table 1:** Percentage of NCs with superstructural vacancy ordering evident in HRTEM images.

**NC diameter**	**NCs with superstructure (%)**
2 nm	0
4 nm	10
6 nm	80

Image analysis was performed on ca. 30 NCs at each size and all images used for analysis are shown in Supplementary Figs 4–6. Superstructural ordering is only observed along the <111> direction; only NCs displaying lattice fringes along this direction were considered in the analysis.

**Table 2:** Crystallographic parameters for simulated diffraction patterns in Fig. 4b.

**6 nm—Cu_2_Se**			Space group: *Fm-*3*m a*_c_=5.85 Å		
**Element**	**Wycoff Pos.**	***x***	***y***	***z***	**Occ.**
Se	4*a*	0	0	0	1
Cu	8*c*	0.25	0.25	0.25	0.37
Cu	32*f*	0.34	0.34	0.34	0.1575
**4 nm—Cu_2_Se**			Space group: *Fm-*3*m a*_c_=5.85 Å		
Se	4*a*	0	0	0	1
Cu	8*c*	0.25	0.25	0.25	1
**2 nm—Cu_2_Se**			Space group: *Fm-*3*m a*_c_=5.85 Å		
Se	4*a*	0	0	0	1
Cu	8*c*	0.25	0.25	0.25	0.3
Cu	4*b*	0.5	0.5	0.5	0.3
Cu	24*e*	0.75	0	0	0.182

**Table 3:** Crystallographic parameters for the simulated diffraction patterns in Fig. 4c.

**4 nm—Cu**_**2**_**Se**			Space group: *Fm-*3*m a*_c_=5.85 Å		
**Element**	**Wycoff pos.**	***x***	***y***	***z***	**Occ.**
Se	4*a*	0	0	0	1
Cu	8*c*	0.25	0.25	0.25	0.37
Cu	32*f*	0.34	0.34	0.34	0.1575
					
**4 nm—Cu**_**2**_**Se**			Space group: *Fm-*3*m a*_c_=5.85 Å		
**Element**	**Wycoff pos.**	***x***	***y***	***z***	**Occ.**
Se	4*a*	0	0	0	1
Cu	8*c*	0.25	0.25	0.25	0.685
Cu	32*f*	0.34	0.34	0.34	0.07875
					
**4 nm—Cu**_**2**_**Se**			Space group: *Fm-*3*m a*_c_=5.85 Å		
**Element**	**Wycoff pos.**	***x***	***y***	***z***	**Occ.**
Se	4*a*	0	0	0	1
Cu	8*c*	0.25	0.25	0.25	1
Cu	32*f*	0.34	0.34	0.34	0

**Table 4:** Crystallographic parameters for the simulated diffraction patterns in Fig. 4d.

**2 nm—Cu**_**2**_**Se**						Space group: *Fm-*3*m a*_c_=5.85 Å				
**Element**	**Wycoff Pos.**			***x***		***y***		***z***		**Occ.**	
Se	4*a*		0		0		0		1	
Cu	8*c*		0.25		0.25		0.25		0.3	
Cu	4*b*		0.5			0.5		0.5		0	
Cu	24*e*			0.25		0		0		0.2333	
											
**2 nm—Cu**_**2**_**Se**					Space group: *Fm-*3*m a*_c_=5.85 Å						
**Element**	**Wycoff Pos.**		***x***		***y***		***z***		**Occ.**		
Se	4*a*		0		0		0		1		
Cu	8*c*		0.25		0.25		0.25		0.3		
Cu	4*b*		0.5		0.5		0.5		0.15		
Cu	24*e*		0.25		0		0		0.2083		
											
**2 nm—Cu**_**2**_**Se**					Space group: *Fm-*3*m a*_c_=5.85 Å						
**Element**	**Wycoff Pos.**		***x***		***y***		***Z***		**Occ.**		
Se	4*a*		0		0		0		1		
Cu	8*c*		0.25		0.25		0.25		0.3		
Cu	4*b*		0.5		0.5		0.5		0.3		
Cu	24*e*		0.25		0		0		0.1833		

## References

[b1] Scholl J. A., Koh A. L. & Dionne J. A. Quantum plasmon resonances of individual metallic nanoparticles. Nature 483, 421–427 (2012). 22437611 10.1038/nature10904

[b2] Alivisatos A. P. Semiconductor cluster, nanocrystals, and quantum dots. Science 271, 933–937 (1996).

[b3] Goldstein A. N., Echer C. M. & Alivisatos A. P. Melting in semiconductor nanocrystals. Science 256, 1425–1427 (1992). 17791609 10.1126/science.256.5062.1425

[b4] Buffat P. & Borel J. P. Size effect on the melting temperature of gold particles. Phys. Rev. A 13, 2287–2298 (1976).

[b5] Rivest J. B., Fong L. K., Jain P. K., Toney M. F. & Alivisatos A. P. Size dependence of a temperature-induced solid–solid phase transition in copper(I) sulfide. J. Phys. Chem. Lett. 2, 2402–2406 (2011).

[b6] Sahu A. . Solid-phase flexibility in Ag_2_Se semiconductor nanocrystals. Nano Lett. 14, 115–121 (2014). 24295334 10.1021/nl4041498

[b7] Makiura R. . Size-controlled stabilization of the superionic phase to room temperature in polymer-coated AgI nanoparticles. Nat. Mater. 8, 476–480 (2009). 19448614 10.1038/nmat2449

[b8] Tolbert S. H. & Alivisatos A. P. Size dependence of a first order solid-solid phase transition: the wurtzite to rock salt transformation in CdSe nanocrystals. Science 265, 373–376 (1994). 17838040 10.1126/science.265.5170.373

[b9] Maier J. Nanoionics: ion transport and electrochemical storage in confined systems. Nat. Mater. 4, 805–815 (2005). 16379070 10.1038/nmat1513

[b10] Lee J. S., Adams S. & Maier J. Transport and phase transition characteristics in AgI: Al_2_O_3_ composite electrolytes: evidence for a highly conducting 7-layer AgI polytype. J. Electrochem. Soc. 147, 2407–2418 (2000).

[b11] Tolbert S. & Alivisatos A. The wurtzite to rock salt structural transformation in CdSe nanocrystals under high pressure. J. Chem. Phys. 201, 4642–4656 (1995).

[b12] Bardhan R. . Uncovering the intrinsic size dependence of hydriding phase transformations in nanocrystals. Nat. Mater. 12, 905–912 (2013). 23913172 10.1038/nmat3716

[b13] Langhammer C., Zhdanov V. P., Zorić I. & Kasemo B. Size-dependent kinetics of hydriding and dehydriding of Pd nanoparticles. Phys. Rev. Lett. 104, 135502 (2010). 20481892 10.1103/PhysRevLett.104.135502

[b14] Danilkin S. A. . Crystal structure and lattice dynamics of superionic copper selenide Cu_2-x_Se. J. Alloys Compd 361, 57–61 (2003).

[b15] Hull S., Keen D. A., Hayes W. & Gardner N. J. G. Superionic behaviour in copper (I) iodide at elevated pressures and temperatures. J. Phys. Condens. Matter 10, 10941–10954 (1999).

[b16] Terabe K., Hasegawa T., Nakayama T. & Aono M. Quantized conductance atomic switch. Nature 433, 47–50 (2005). 15635405 10.1038/nature03190

[b17] Kasuya A. . Ultra-stable nanoparticles of CdSe revealed from mass spectrometry. Nat. Mater. 3, 99–102 (2004). 14743211 10.1038/nmat1056

[b18] Beecher A. N. . Atomic structures and gram scale synthesis of three tetrahedral quantum dots. J. Am. Chem. Soc. 136, 10645–10653 (2014). 25003618 10.1021/ja503590h

[b19] Yu K. . Thermodynamic equilibrium-driven formation of single-sized nanocrystals: reaction media tuning CdSe magic-sized versus regular quantum dots. J. Phys. Chem. C 114, 3329–3339 (2010).

[b20] Jain P. K., Amirav L., Aloni S. & Alivisatos A. P. Nanoheterostructure cation exchange: anionic framework conservation. J. Am. Chem. Soc. 132, 9997–9999 (2010). 20593896 10.1021/ja104126u

[b21] Li H. . Sequential cation exchange in nanocrystals: preservation of crystal phase and formation of metastable phases. Nano Lett. 11, 4964–4970 (2011). 21961554 10.1021/nl202927a

[b22] Luther J. M., Jain P. K., Ewers T. & Alivisatos A. P. Localized surface plasmon resonances arising from free carriers in doped quantum dots. Nat. Mater. 10, 361–366 (2011). 21478881 10.1038/nmat3004

[b23] Dorfs D. . Reversible tunability of the near-infrared valence band plasmon resonance in Cu_2−x_Se nanocrystals. J. Am. Chem. Soc. 133, 11175–11180 (2011). 21728384 10.1021/ja2016284

[b24] Bekenstein Y. . Thermal doping by vacancy formation in copper sulfide nanocrystal arrays. Nano Lett. 14, 1349–1353 (2014). 24564833 10.1021/nl4043642

[b25] Hsu S. W., Bryks W. & Tao A. R. Effects of carrier density and shape on the localized surface plasmon resonances of Cu_2–x_S nanodisks. Chem. Mater. 24, 3765–3771 (2012).

[b26] Kriegel I. . Tuning the excitonic and plasmonic properties of copper chalcogenide nanocrystals. J. Am. Chem. Soc. 134, 1583–1590 (2012). 22148506 10.1021/ja207798q

[b27] Jain P. K. Plasmon-in-a-Box: on the physical nature of few-carrier plasmon resonances. J. Phys. Chem. Lett. 5, 3112–3119 (2014). 26276321 10.1021/jz501456t

[b28] Jain P. K. . Doped nanocrystals as plasmonic probes of redox chemistry. Angew. Chem. Int. Ed. 52, 13671–13675 (2013). 10.1002/anie.201303707 24155083

[b29] Kashida S. & Akai J. X-ray diffraction and electron microscopy studies of the room-temperature structure of Cu_2_Se. J. Phys. C Solid State Phys. 21, 5329–5336 (1988).

[b30] Liu H. . Copper ion liquid-like thermoelectrics. Nat. Mater. 11, 422–425 (2012). 22406814 10.1038/nmat3273

[b31] Milat O., Vučić Z. & Rušćić B. Superstructural ordering in low-temperature phase of superionic Cu_2_Se. Solid State Ion. 23, 37–47 (1987).

[b32] Hu T., Wittenberg J. S. & Lindenberg A. M. Room-temperature stabilization of nanoscale superionic Ag_2_Se. Nanotechnology 25, 415705 (2014). 25249347 10.1088/0957-4484/25/41/415705

[b33] Heyding R. D. & Murray R. M. The crystal structures of Cu_1.8_Se, Cu_3_Se_2_ , α- and γCuSe, CuSe_2_, and CuSe_2_II. Can. J. Chem. 54, 841–848 (1976).

[b34] Yamamoto K. & Kashida S. X-ray study of the cation distribution in Cu_2_Se, Cu_1.8_Se and Cu_1.8_S; analysis by the maximum entropy method. Solid State Ion. 48, 241–248 (1991).

[b35] Yamamoto K. & Kashida S. X-ray study of the average structures of Cu_2_Se and Cu_1.8_S in the room temperature and the high temperature phases. J. Solid State Chem. 93, 202–211 (1991).

[b36] Wang Y. . Design principles for solid-state lithium superionic conductors. Nat. Mater. 14, 1026–1031 (2015). 26280225 10.1038/nmat4369

[b37] Boyce J. B., Hayes T. M. & Mikkelsen J. C. EXAFS investigation of mobile-ion density: CuI and Cu_2_Se contrasted. Solid State Ion. 5, 497–500 (1981).

[b38] Phillips J. C. Covalent-ionic and covalent-metallic transitions of tetrahedrally coordinated A_N_B_8−N_ crystals under pressure. Phys. Rev. Lett. 27, 8–11 (1971).

[b39] Ishii M., Shibata K. & Nozaki H. Anion distributions and phase transitions in CuS_1−x_Se_x_(x=0–1) studied by Raman spectroscopy. J. Solid State Chem. 105, 504–511 (1993).

[b40] Sneed B. T., Young A. P. & Tsung C. K. Building up strain in colloidal metal nanoparticle catalysts. Nanoscale 7, 12248–12265 (2015). 26147486 10.1039/c5nr02529j

[b41] Richter H., Wang Z. P. & Ley L. The one phonon Raman spectrum in microcrystalline silicon. Solid State Commun. 39, 625–629 (1981).

[b42] Scamarcio G., Lugar M. & Manno D. Size-dependent lattice contraction in CdS_1−x_Se_x_ nanocrystals embedded in glass observed by Raman scattering. Phys. Rev. B 45, 13792–13795 (1992). 10.1103/physrevb.45.13792 10001487

[b43] Milman V. Klockmannite, CuSe: structure, properties and phase stability from *ab initio* modeling. Acta Crystallogr. B B58, 437–447 (2002). 10.1107/s0108768102003269 12037331

[b44] Tolbert S. & Alivisatos A. P. The wurtzite to rock salt structural transformation in CdSe nanocrystals under high pressure. J. Chem. Phys. 102, 4642–4656 (1995).

[b45] Sun J., Clark B. K., Torquato S. & Car R. The phase diagram of high-pressure superionic ice. Nat. Commun. 6, 8156 (2015). 26315260 10.1038/ncomms9156 PMC4560814

[b46] Miller T. A. . The mechanism of ultrafast structural switching in superionic copper (I) sulphide nanocrystals. Nat. Commun. 4, 1369 (2013). 23340409 10.1038/ncomms2385

[b47] Yang Y. A., Wu H., Williams K. R. & Cao Y. C. Synthesis of CdSe and CdTe nanocrystals without precursor injection. Angew. Chem. Int. Ed. 44, 6712–6715 (2005). 10.1002/anie.200502279 16187382

[b48] Carbone L. . Synthesis and micrometer-scale assembly of colloidal CdSe/CdS nanorods prepared by a seeded growth approach. Nano Lett. 7, 2942–2950 (2007). 17845067 10.1021/nl0717661

[b49] Liu L. . Shape control of CdSe nanocrystals with zinc blende structure. J. Am. Chem. Soc. 131, 16423–16429 (2009). 19902978 10.1021/ja903633d

[b50] Deka S. . Phosphine-free synthesis of p-type copper(I) selenide nanocrystals in hot coordinating solvents. J. Am. Chem. Soc. 132, 8912–8914 (2010). 20540521 10.1021/ja103223x

