# Supplementary material for: Liquid-like cationic sub-lattice in copper selenide clusters
Source: Nat Commun. 2017 Feb 20;8:14514. doi: 10.1038/ncomms14514 (PMC5321727; doi:10.1038/ncomms14514)
Supplement: Supplementary Information — Supplementary Figures and Supplementary References. [file ncomms14514-s1.pdf]

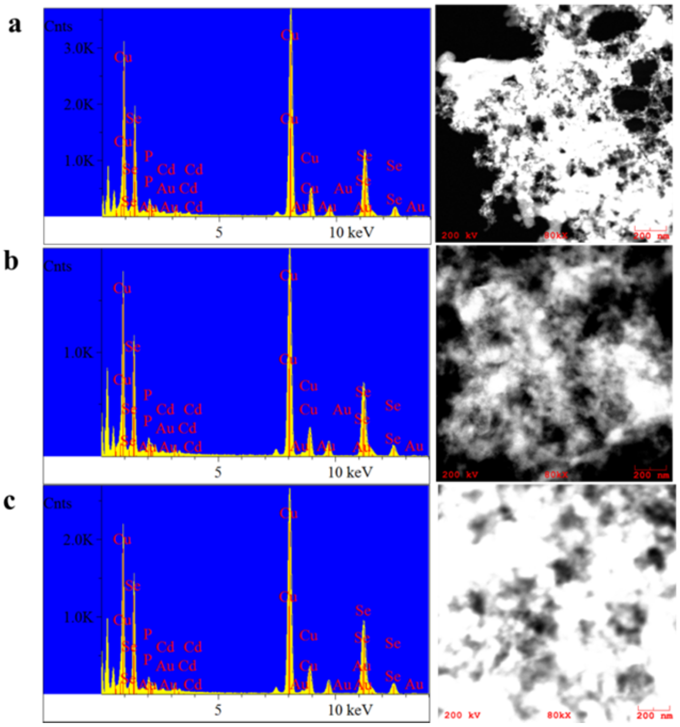

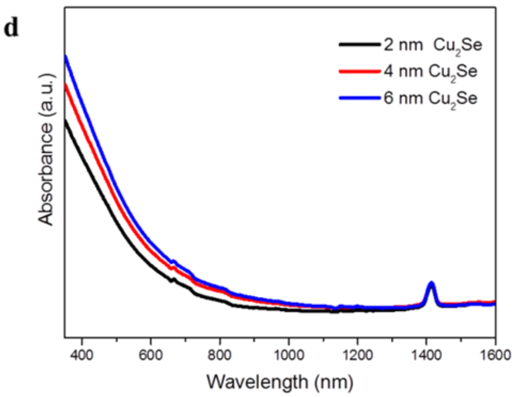

e

NC Diameter	Cu: Se: Cd Atomic %	Stoichiometry
2 nm	2.28: 1: 0.09	$Cu_{2.2}Se$
4 nm	2.11: 1: 0.09	$Cu_{2.1}Se$
6 nm	2.05: 1: 0.09	$Cu_{2.04}Se$

Supplementary Figure 1. Energy-dispersive X-ray spectroscopy (EDS) of a wide-field of a) 2 nm, b) 4 nm and c) 6 nm Cu_2Se nanocrystals (NCs), respectively. To the right of each spectrum us shown a low-magnification dark-field scanning transmision electron microscopy (STEM) image of the wide-field over which the EDS was measured. All STEM image scale bars correspond to 200 nm. Samples were prepared by cation exchange of 2 nm, 4 nm, and 6 nm zincblende CdSe nanocrystals in toluene with a methanolic solution of Cu^+, as described in the Methods section. Exchange to Cu_2Se was monitored via UV-Vis absorption spectrophotometry. Panel d) shows the absorption spectra for the final 2 nm, 4 nm, and 6 nm Cu_2Se NCs subject to EDS analysis. Following exchange, samples were precipitated with methanol, dispersed in toluene, drop-cast onto an ultrathin carbon Au-mesh TEM grid, and washed with methanol several times. STEM/EDS was performed on a JEOL 2010F instrument operated at 200 kV with a 0.5-nm size beam. Panel e) shows a table of the elemental compositions and stoichiometries for the 2 nm, 4 nm, and 6 nm Cu_2Se NCs as determined by EDS. The Cu:Se ratio is ca. 2:1 for all three sizes. In the EDS spectra, the Cd signal is at the noise level of the measurement. The presence of Au signals is due to the Au-mesh of the TEM grids.

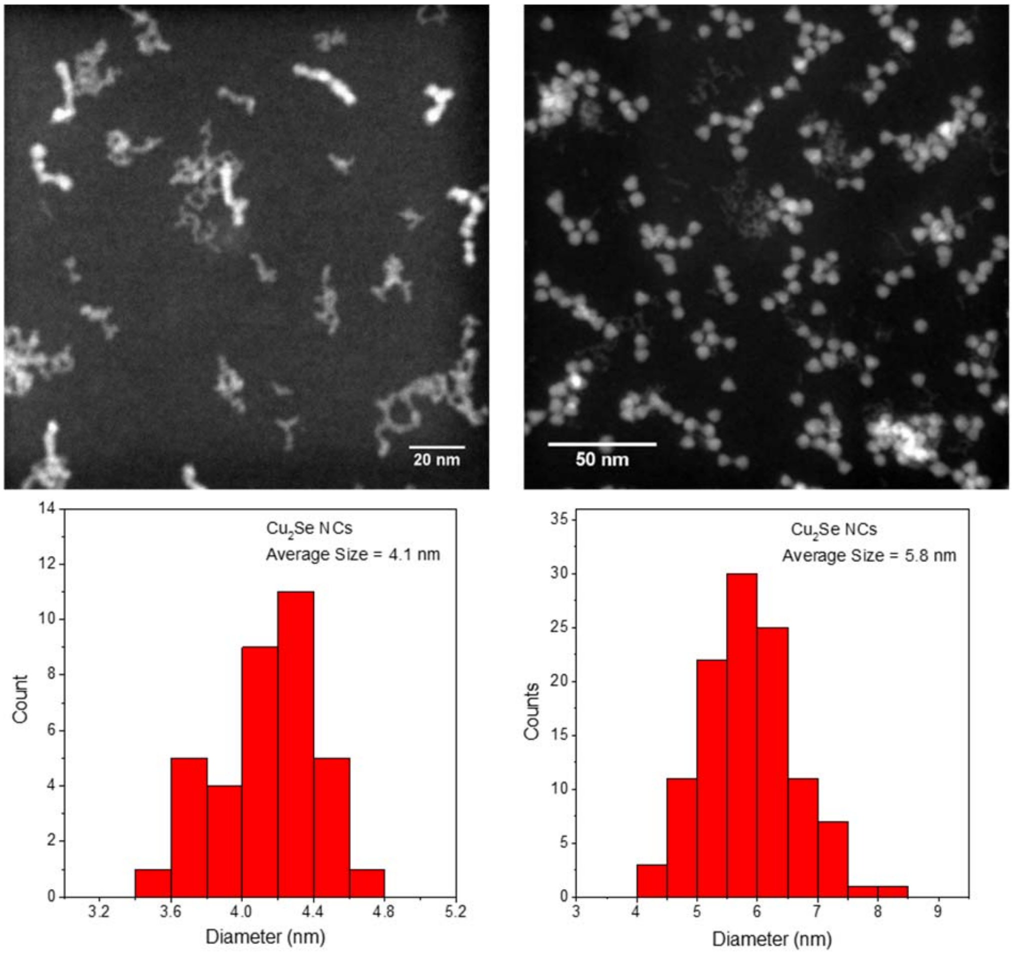

Supplementary Figure 2. High-angle annular dark field scanning transmission electron microscopy (HAADF-STEM) of 4 nm and 6 nm Cu_2Se nanocrystals (NCs). Top panel shows representative HAADF-STEM images for 4 nm and 6 nm NCs obtained from cation exchange. NC sizes were estimated manually in Image J from STEM images. The diameter of the quasi-spherical NC domains was measured using the line tool. Data from ca. 40 NC domains was used to make the size histograms shown in the bottom panel and to determine average diameters. Samples were prepared by titrating solutions of 4 nm and 6 nm zincblende CdSe nanocrystals in toluene with a methanolic solution of Cu^+, as described in the Methods section. Exchange to Cu_2Se was monitored via UV-Vis absorption spectroscopy. Fully exchanged samples were precipitated with methanol, dispersed in toluene, drop-cast onto an ultrathin carbon TEM grid, and washed with methanol several times. HAADF-STEM images were acquired on a JEOL 2010F at 200 kV with a 0.5-nm size beam.

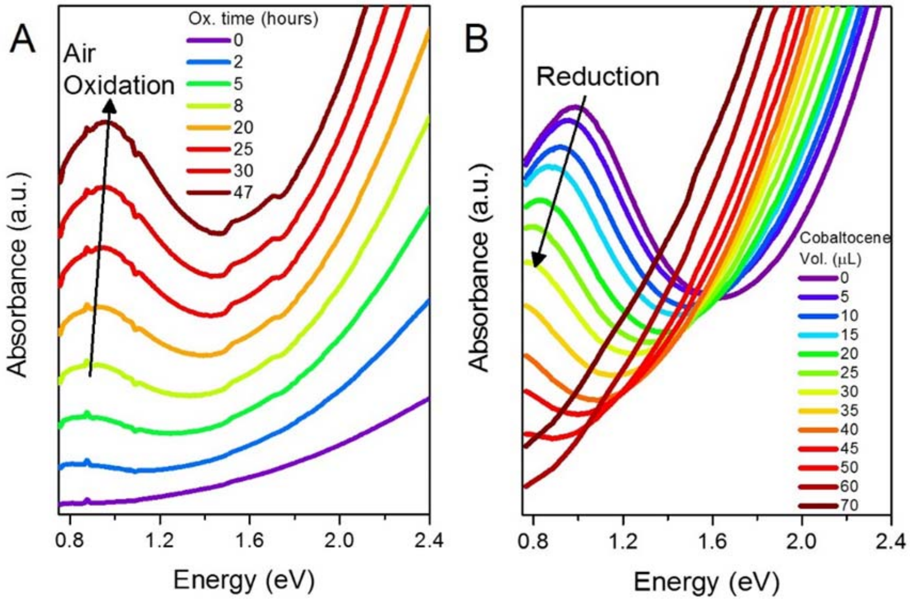

Supplementary Figure 3. A) Ultraviolet-visible absorption spectra of 6 nm Cu_2Se nanocrystals (NCs) in the course of oxidation in air. Air oxidation results in the removal of Cu in the form of copper oxide, leading to formation of holes in the valence band. Hole formation is manifested in the emergence of a localized surface plasmon resonance (LSPR) band. With increasing oxidation time, the LSPR band blue-shifts, signifying an increase in the hole density. The peak energy of the LSPR eventually stabilizes to a value of 0.99 eV. B) UV-vis absorption spectra of the oxidized 6 nm Cu_2Se NCs in the course of titration with a solution of the reducing agent cobaltocene. Cobaltocene injects electrons into the NCs annihilating the holes in the valence band. As a result, the LSPR redshifts and decreases in intensity, signifying a decrease in the hole density. The 6 nm Cu_2Se NCs were prepared by the cation exchange of 6 nm wurtzite CdSe NCs.

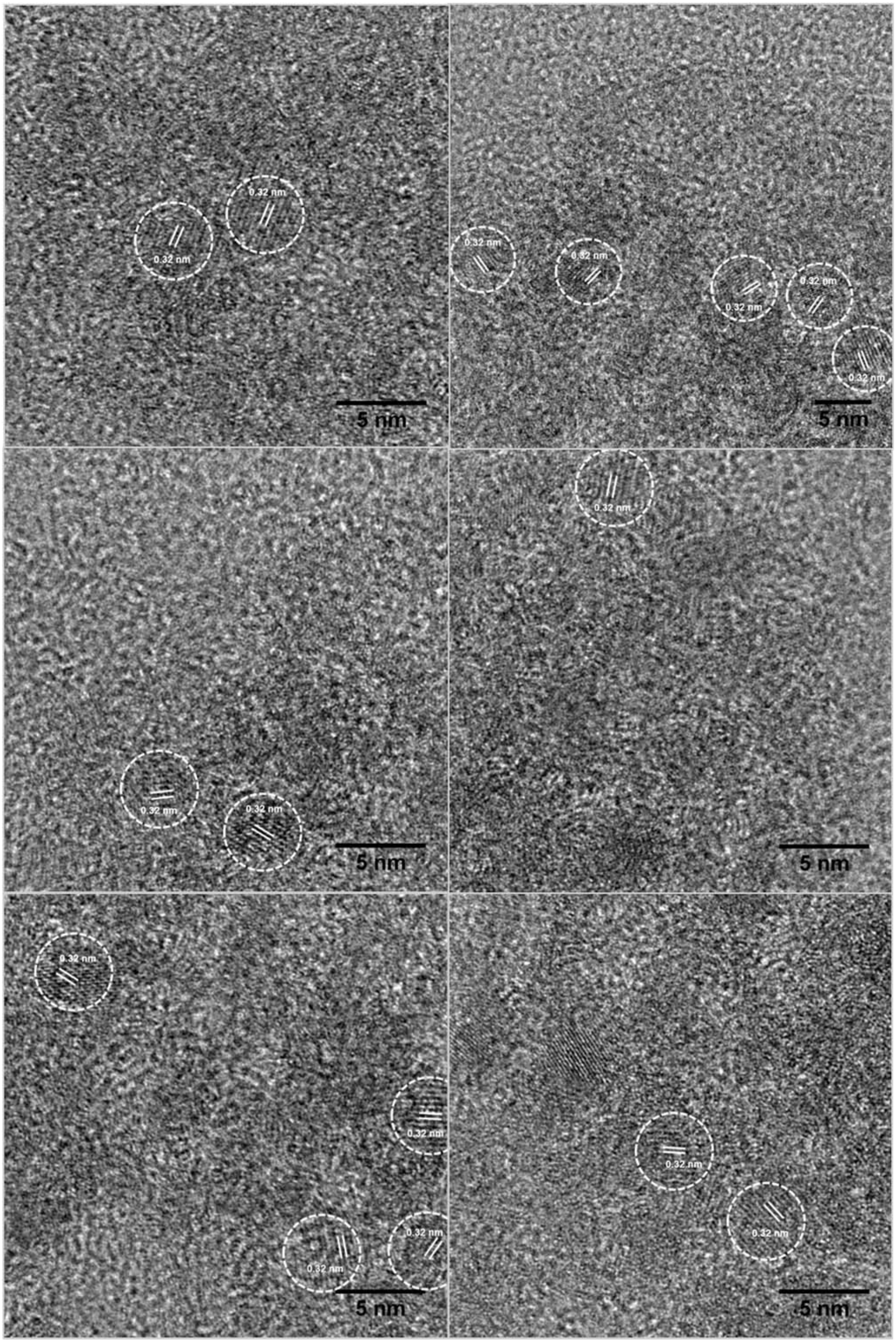
0.32 nm
5 nm

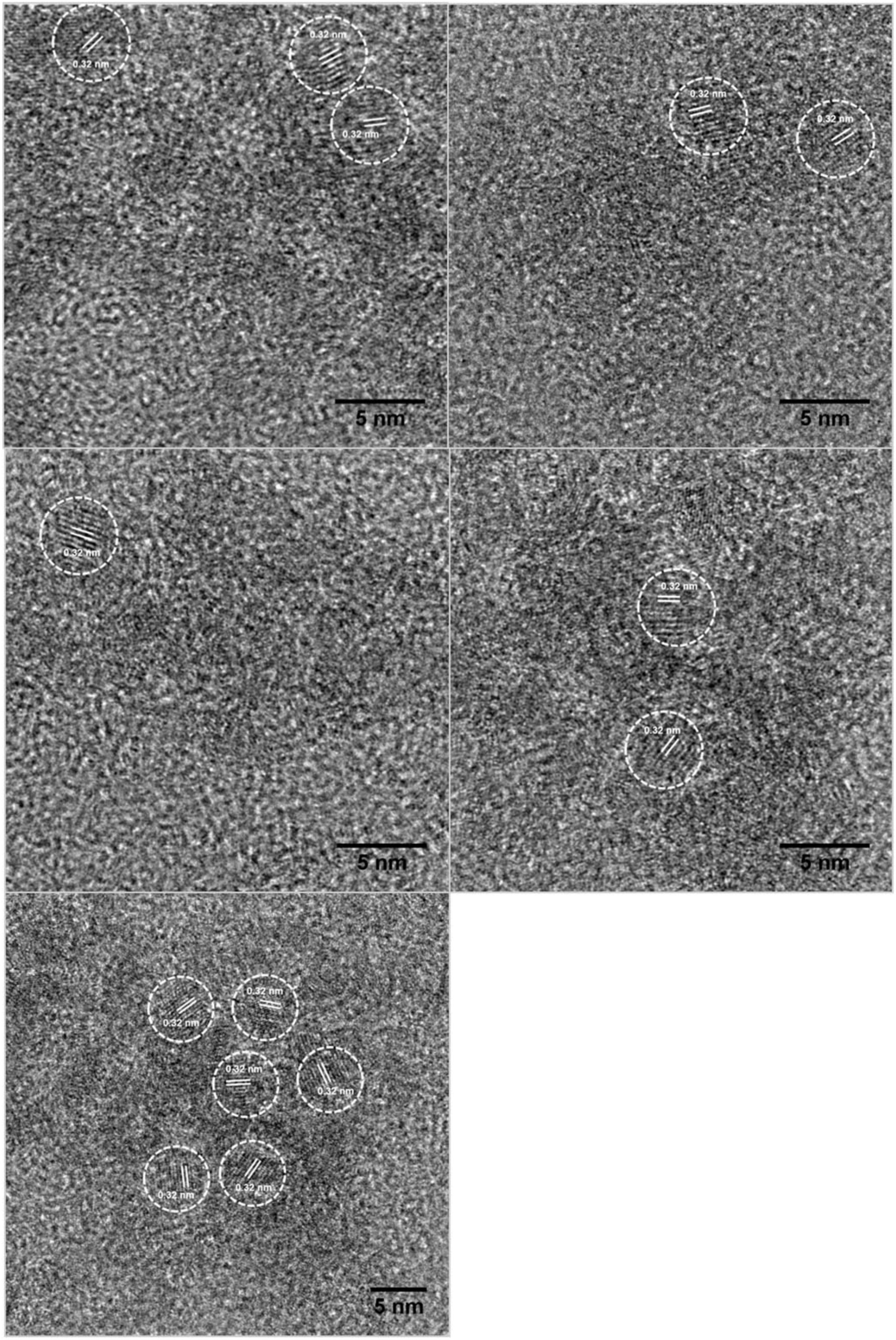
0.32 nm
5 nm

Supplementary Figure 4. High resolution transmission electron microscopy (HRTEM) images of several more 2 nm Cu_2Se clusters. Measured lattice spacings between {111} planes are shown for many of the clusters in the wide-field images. All scale bars correspond to 5 nm.

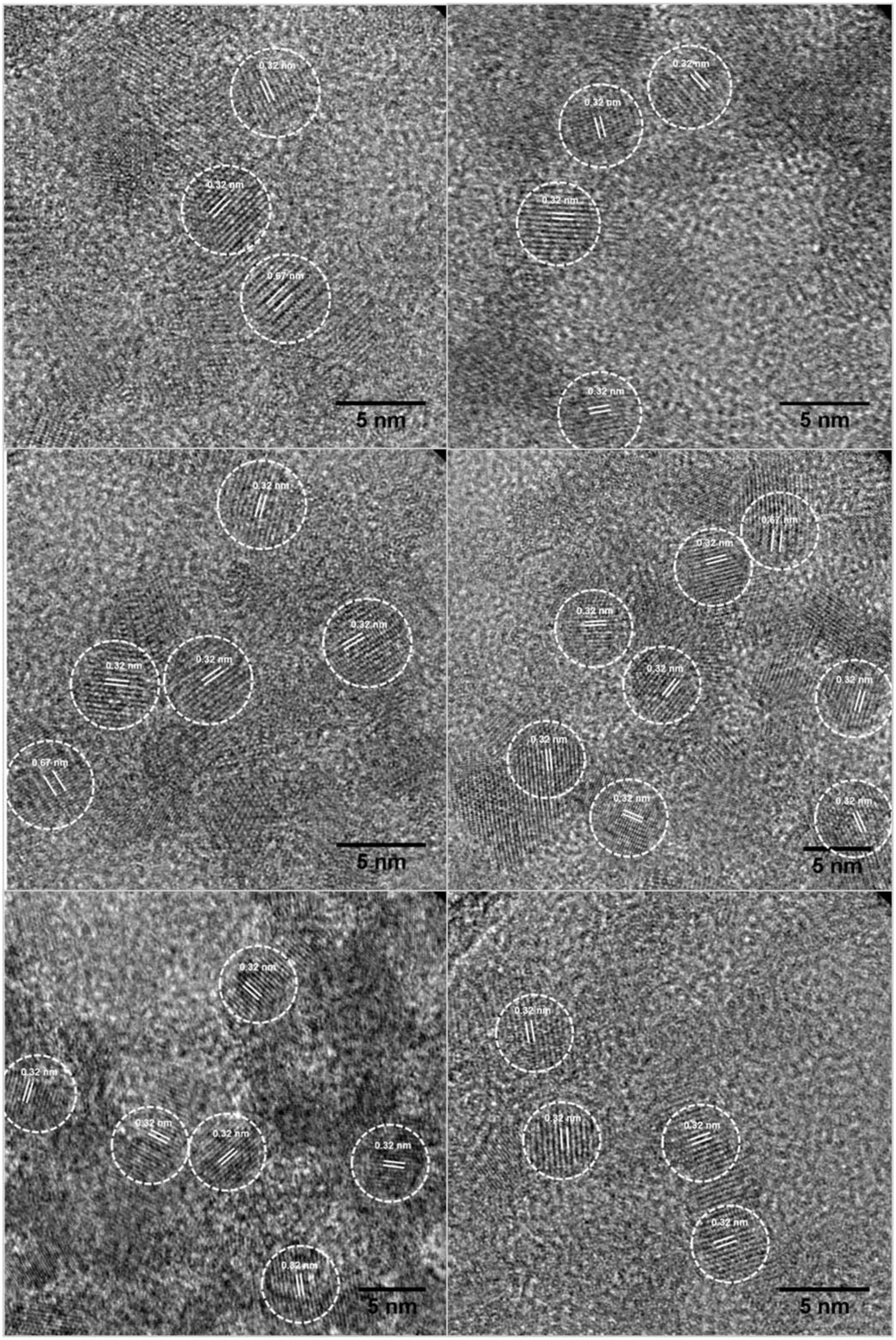

0.32 nm
0.32 nm
0.67 nm
5 nm
0.32 nm
0.32 nm
0.32 nm
0.32 nm
5 nm
0.32 nm
0.32 nm
0.32 nm
0.32 nm
0.67 nm
5 nm
0.67 nm
0.32 nm
0.32 nm
0.32 nm
0.32 nm
0.32 nm
0.32 nm
0.32 nm
5 nm
0.32 nm
0.32 nm
0.32 nm
0.32 nm
0.32 nm
0.32 nm
5 nm
0.32 nm
0.32 nm
0.32 nm
0.32 nm
5 nm

Supplementary Figure 5. High resolution transmission electron microscopy (HRTEM) images of several more 4 nm Cu_2Se nanocrystals (NCs). Measured lattice spacings between {111} planes are shown for many of the NCs in the wide-field images. All scale bars correspond to 5 nm.

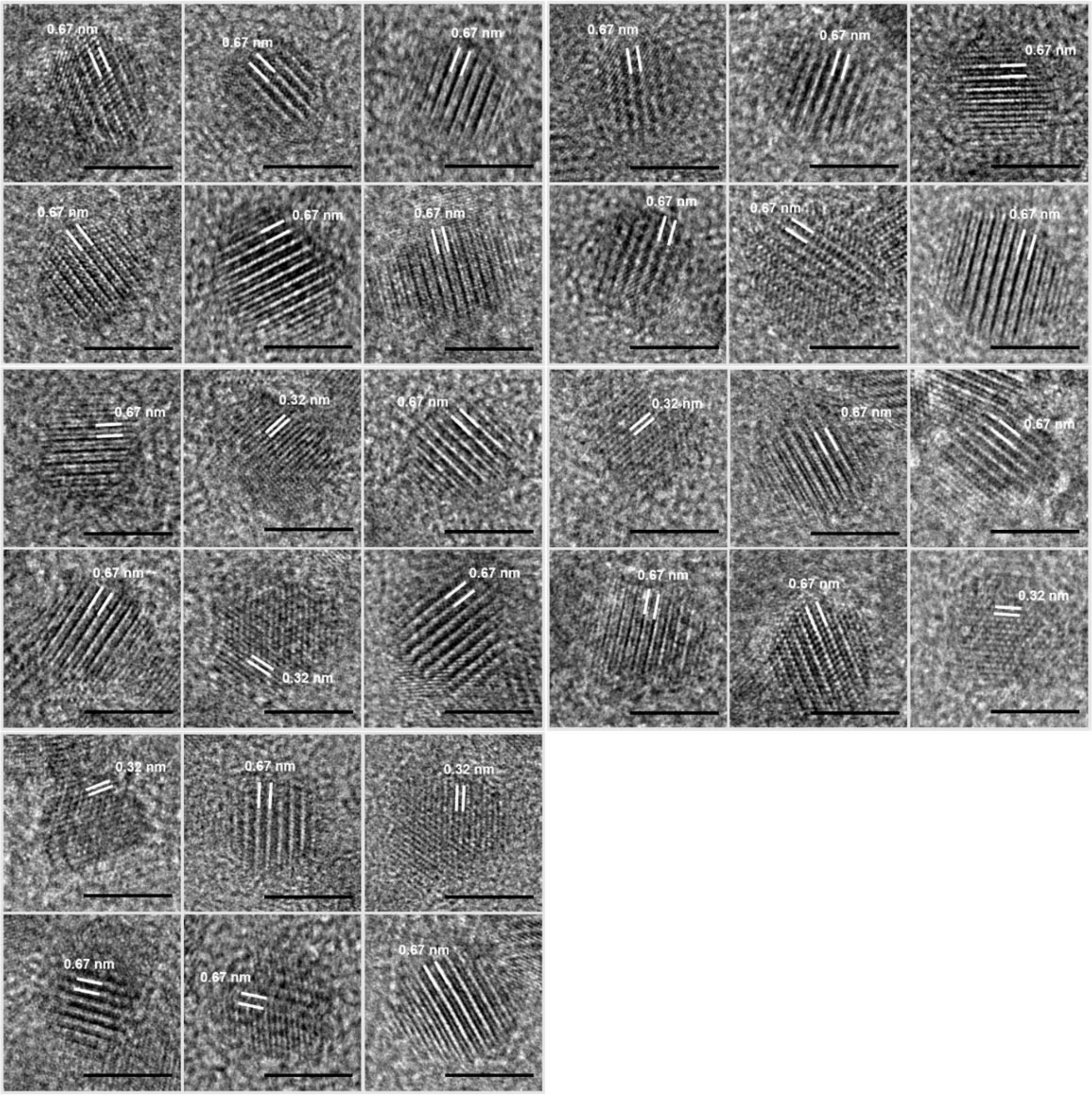

Supplementary Figure 6. High resolution transmission electron microscopy (HRTEM) images of several more 6 nm Cu_2Se nanocrystals (NCs). Measured lattice spacings between {111} planes are shown for all NCs. All scale bars correspond to 5 nm.

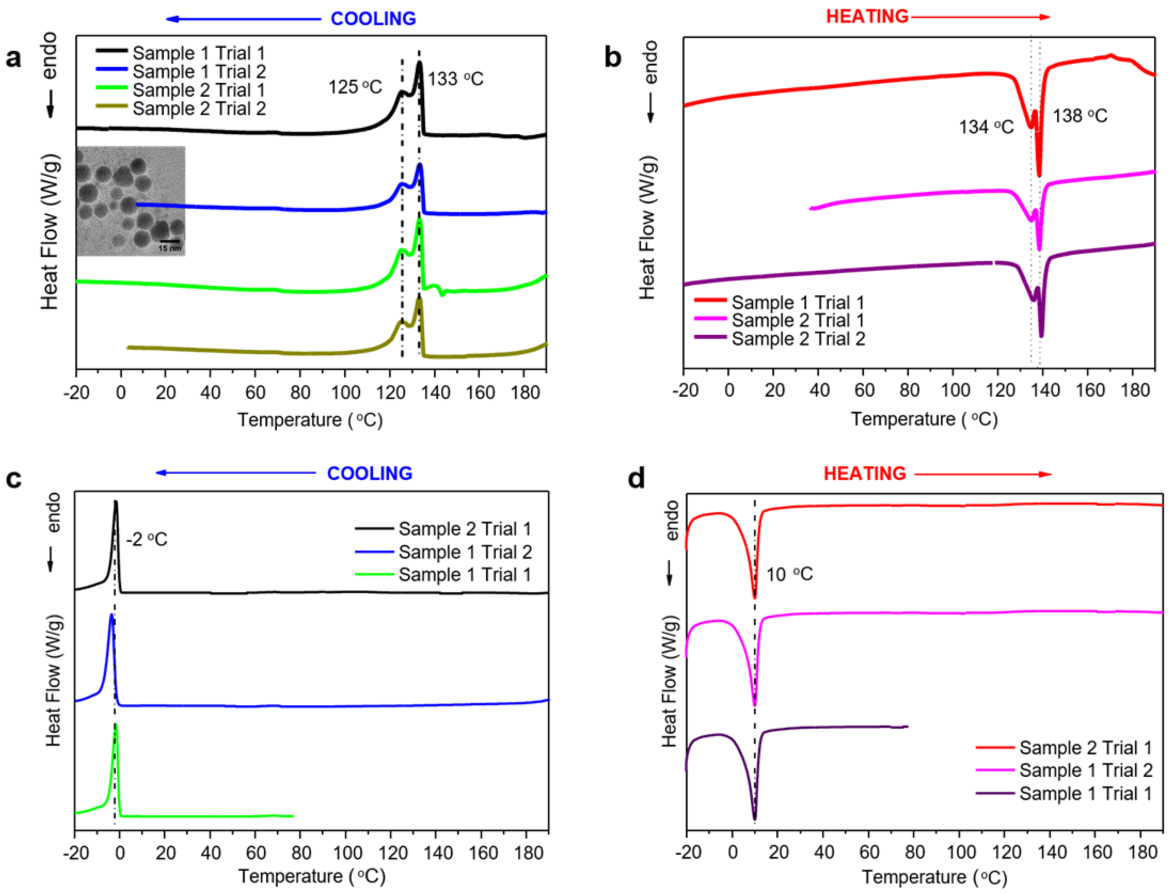

Supplementary Figure 7. Differential scanning calorimetry (DSC) thermograms for larger-size Cu_2Se nanocrystals (NCs) (a-cooling, b-heating) as compared to those for 2 nm Cu_2Se clusters (c-cooling, d-heating), respectively. Upon heating, the larger-size NCs show endothermic peaks at 134-138 ^{o}C, corresponding to the β→α transition. Upon cooling, exothermic peaks are obtained at 125-133 ^{o}C, indicating some degree of hysterisis. The 2 nm clusters, upon heating, show an endothermic peak at 10 ^{o}C, corresponding to the β→α transition. Upon cooling, an exothermic peaks is obtained at -2 ^{o}C, indicating some degree of hysterisis. For ensuring reproducibility, at each size, thermograms were measured for two separate samples. For atleast one sample at each size, the DSC scan was run twice and confirmed to be repeatable. The larger NCs were prepared as per the procedure of Deka et. al.,[1] precipitated with methanol, dispersed in toluene, and then drop-cast onto an ultrathin carbon TEM grid, which was washed with methanol several times prior to imaging. High resolution transmission electron microscopy (HRTEM) imaging was performed on a JEOL 2010F instrument operated at 200 kV with a 0.5-nm size beam.The inset in panel a) shows a representative HRTEM image of the larger NC sample showing NCs in the 9-16 nm size range (scale bar = 15 nm).

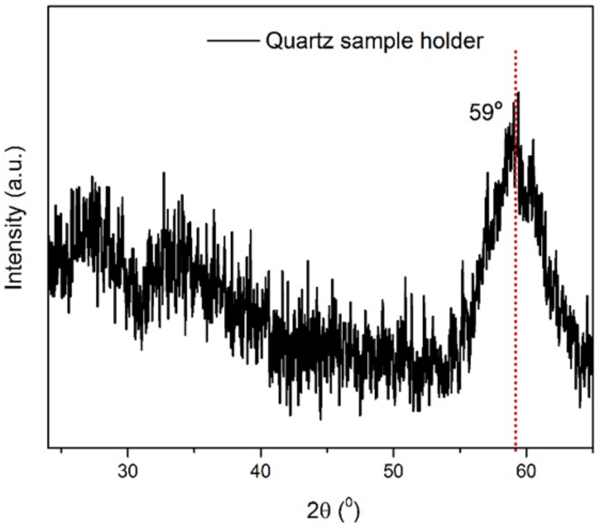

Supplementary Figure 8. The peak at $2\theta = 59°$ in our diffraction patterns is from the quartz sample holder, as seen from a pattern of the holder without any sample. Note in Figure 4a, this sample-holder peak appears to increase in intensity, relative to the sample nanocrystal (NC) diffraction peaks, as we go to smaller NC size. This is simply a result of the reduced intensity of the diffraction peaks for the smaller NC samples.

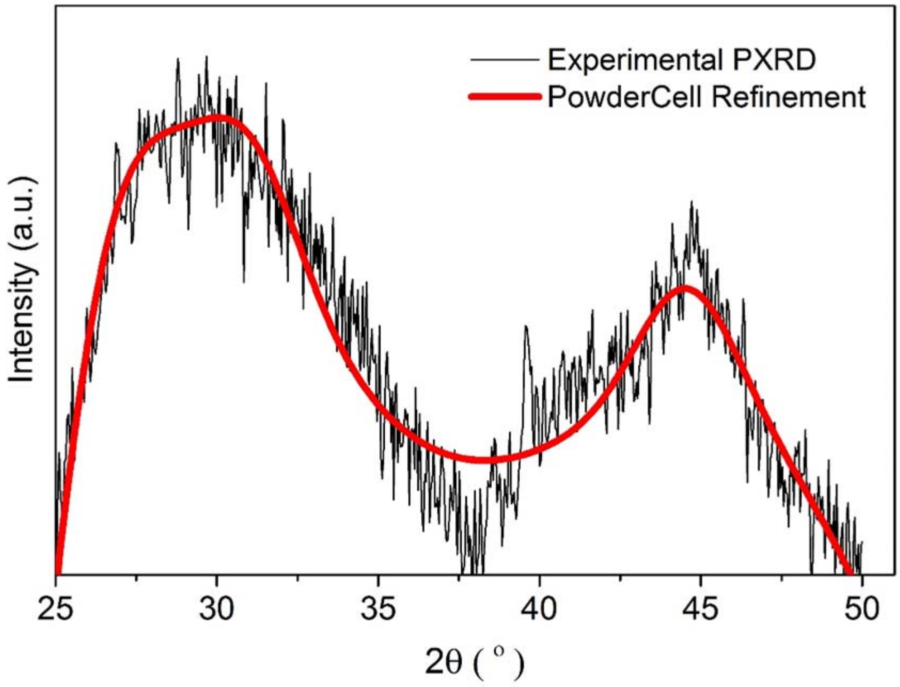

Supplementary Figure 9. The experimental powder X-ray diffraction (PXRD) pattern for the 2 nm clusters (background removed, 2θ = 25-50° range) was subject to Rietveld refinement in Powder Cell using 5th order polynomial fitting. The fit (R_p =11.52, R_{wp} =13.32) is shown overlaying the scaled experimental pattern. The starting unit cell structure was based on parameters for the 2 nm clusters from Table 2. The lattice constant a_c, scale factor, U, V, W, and Cu site occupancy factors (SOFs) in 4*b* and 8*c* sites were allowed to vary. The Se SOF in 4*a* sites was set to 1 and Cu SOF in 24*e* sites was set to 0.182. The result of the fitting yielded a_c = 5.75 Å, SOF for 4*b* = 0.36, and SOF for 8*c* = 0.28, i.e., 18% Cu^+ occupancy in octahedral sites and 28% occupancy in tetrahedral sites with remaining in 24*e* sites. Note that there are uncertainties involved in this determination due to the low signal-to-noise ratio (S/N) of the experimental pattern of the 2 nm clusters coupled with severe peak broadening and overlap. More rigorous Rietveld analysis and structure determination will be possible using PXRD or full scattering data obtained at a synchrotron. Synchrotron data is expected to have high S/N and low X-ray fluorescence background.

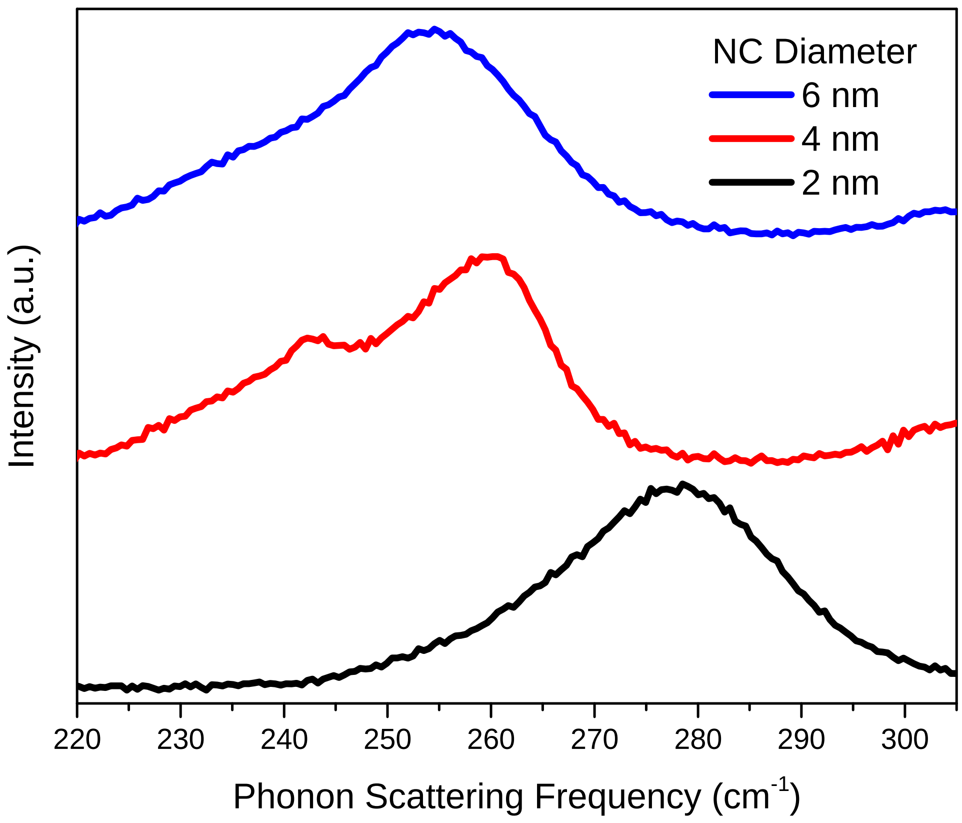

Supplementary Figure 10. Representative phonon scattering spectra of samples of 2 nm Cu_2Se clusters, 4 nm Cu_2Se NCs, and 6 nm Cu_2Se nanocrystals (NCs) in dreid film form on a Si substrate. The primary band seen in each spectrum corresponds to the A_1 longitudinal optical (LO) phonon mode of the Cu_2Se lattice.The frequency of the LO mode increases with decreasing NC size due to the effect of compressive strain. This effect is plotted in Figure 5 in the main text. Spectra plotted were subject to baseline subtraction and intensity scaling to facilitate easy comparison. Cu_2Se clusters and NCs were prepared by cation exchange of zincblende CdSe clusters and NCs of the corresponding size.

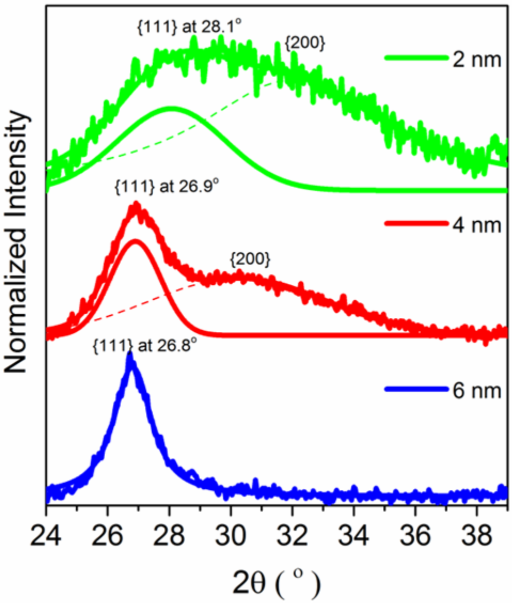

NC size D (nm)	$2\theta_{111}$ (°)	$\sin\theta$ (λ=1.54 Å)	d_{111} (Å)	% strain (relative to 6 nm)
2	28.1	0.2426	3.17	-4.5
4	26.9	0.2325	3.31	-0.4
6	26.8	0.2316	3.32	0.0

Supplementary Figure 11. Powder X-ray diffraction (PXRD) patterns were analyzed for determination of lattice strain as a function of nanocrystal (NC) size. The experimental patterns for all three size NCs in the range from $2\theta = 24$ to 39° were fit to multi-peak Voigt functions (two peaks for the 2 nm and 4 nm sample patterns and one peak for the 6 nm sample pattern). The peak position for the {111} reflection, identified from the fit, is indicated for each size. The strain in the 2 nm clusters relative to the 6 nm NCs is:

$$\frac{\Delta d_{111}}{d_{111}} = -0.045 \quad (1)$$

which implies a compressive strain of 4.5%. A table on the right summarizes the results of the strain estimation from the {111} peak. Note that strain may not be isotropic.

SUPPLEMENTARY REFERENCES

1. Deka, S. *et al.* Phosphine-free synthesis of p-type copper(I) selenide nanocrystals in hot coordinating solvents, *J. Am. Chem. Soc.* **132**, 8912–8914 (2010).
